# Aptamer binding footprints discriminate α-synuclein fibrillar polymorphs from different synucleinopathies

**DOI:** 10.1093/nar/gkae544

**Published:** 2024-06-25

**Authors:** Alix Bouvier-Müller, Deborah Fourmy, Alexis Fenyi, Luc Bousset, Ronald Melki, Frédéric Ducongé

**Affiliations:** CEA, DRF, Institut of biology JACOB, Molecular Imaging Research Center (MIRCen), Fontenay aux roses 92335, France; CNRS UMR 9199, Laboratoire des Maladies Neurodégénératives, Fontenay aux roses 92335, France; Université Paris-Saclay, Fontenay aux roses 92335, France; CEA, DRF, Institut of biology JACOB, Molecular Imaging Research Center (MIRCen), Fontenay aux roses 92335, France; CNRS UMR 9199, Laboratoire des Maladies Neurodégénératives, Fontenay aux roses 92335, France; Université Paris-Saclay, Fontenay aux roses 92335, France; CEA, DRF, Institut of biology JACOB, Molecular Imaging Research Center (MIRCen), Fontenay aux roses 92335, France; CNRS UMR 9199, Laboratoire des Maladies Neurodégénératives, Fontenay aux roses 92335, France; Université Paris-Saclay, Fontenay aux roses 92335, France; CEA, DRF, Institut of biology JACOB, Molecular Imaging Research Center (MIRCen), Fontenay aux roses 92335, France; CNRS UMR 9199, Laboratoire des Maladies Neurodégénératives, Fontenay aux roses 92335, France; Université Paris-Saclay, Fontenay aux roses 92335, France; CEA, DRF, Institut of biology JACOB, Molecular Imaging Research Center (MIRCen), Fontenay aux roses 92335, France; CNRS UMR 9199, Laboratoire des Maladies Neurodégénératives, Fontenay aux roses 92335, France; Université Paris-Saclay, Fontenay aux roses 92335, France; CEA, DRF, Institut of biology JACOB, Molecular Imaging Research Center (MIRCen), Fontenay aux roses 92335, France; CNRS UMR 9199, Laboratoire des Maladies Neurodégénératives, Fontenay aux roses 92335, France; Université Paris-Saclay, Fontenay aux roses 92335, France

## Abstract

Synucleinopathies, including dementia with Lewy bodies (DLB), Parkinson's disease (PD), and multiple system atrophy (MSA), are characterized by the presence of α-synuclein (α-syn) aggregates in the central nervous system. Recent evidence suggests that the heterogeneity of synucleinopathies may be partly explained by the fact that patients may have different α-syn fibrillar polymorphs with structural differences. In this study, we identify nuclease resistant 2′fluoro-pyrimidine RNA aptamers that can differentially bind to structurally distinct α-syn fibrillar polymorphs. Moreover, we introduce a method, AptaFOOT-Seq, designed to rapidly assess the affinity of a mixture of these aptamers for different α-SYN fibrillar polymorphs using next-generation sequencing. Our findings reveal that the binding behavior of aptamers can be very different when they are tested separately or in the presence of other aptamers. In this case, competition and cooperation can occur, providing a higher level of information, which can be exploited to obtain specific ‘footprints’ for different α-Syn fibrillar polymorphs. Notably, these footprints can distinguish polymorphs obtained from patients with PD, DLB or MSA. This result suggests that aptaFOOT-Seq could be used for the detection of misfolded or abnormal protein conformations to improve the diagnosis of synucleinopathies.

## Graphical Abstract

**Graphical Abstract:**
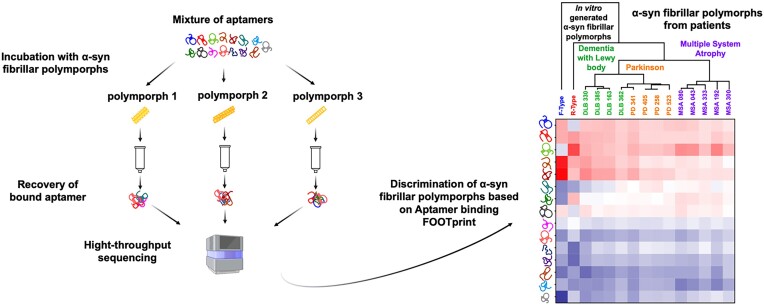

## Introduction

Synucleinopathies are a group of neurodegenerative diseases that include dementia with Lewy bodies (DLB), Parkinson's disease (PD), and multiple system atrophy (MSA) (1). Distinguishing the different synucleinopathies from each other and from other neurodegenerative diseases remains a major challenge, mainly because patients share many common symptoms, especially in the early stages of the disease. For example, although DLB is the second most common cause of neurodegenerative disease, it is often misdiagnosed as Alzheimer's disease or a psychiatric disorder, especially in its early stages. In 2020, a survey of >2000 people with Parkinson's disease (PD) has also found that 26% of people with PD were first told they suffered from another disease (https://www.parkinsons.org.uk/news/poll-finds-quarter-people-parkinsons-are-wrongly-diagnosed). Of those who were misdiagnosed, 48% were treated for a condition that did not exist and 34% declared worsening of their clinical symptoms as a result. In addition, comparative studies between clinical diagnoses and post-mortem neuropathological examination of the brain, which provides the ultimate diagnostic certainty, show that around 20% of patients with DLB features are misdiagnosed (2). There is therefore an urgent need to improve the early and accurate diagnosis of distinct synucleinopathies to implement suited therapies and develop innovative therapies.

The pathological hallmark of synucleinopathies is the progressive accumulation of misfolded, aggregated α-synuclein (α-syn) in the central nervous system (1,3). Beta-strand-rich α-syn domains can stack into fibrillar assemblies that can elongate by recruiting monomeric α-syn (4,5). Such aggregates exhibit different appearances within neuronal cells, and develop in selective regions of the brain and in peripheral nervous tissue, depending on the disease. The inclusions are predominantly neuronal and are termed Lewy bodies and Lewy neurites in PD and DLB (6,7). In contrast, in MSA, the inclusions are also located in oligodendrocytes and Schwann cells (8). In all synucleinopathies, α-syn inclusions gradually affect more and more areas of the nervous system with disease progression, but at different rates and with different symptoms (9).

It has been proposed that α-syn aggregates spread in the brain in a prion-like manner (4). The prion-like behavior of α-syn assemblies has been studied *in vitro* and *in vivo* (10–13). It has been shown that aggregated forms of α-syn can induce the conversion of endogenous α-syn to the pathological form, which can spread throughout the brain, propagating protein misfolding and disease. In addition, it has been proven that recombinant α-syn can assemble *in vitro* into different fibrillar polymorphs that can be distinguished based on their shape, beta sheet content, proteolytic patterns or physical properties (14–17). Most importantly, these different polymorphs can promote different types of neuropathology when injected *in vivo* into the brain or peripheral organs of healthy animals (12,18,19). These results have led to the hypothesis that different α-syn fibrillar polymorphs or ‘strains’ may be present or even responsible for the different clinicopathological features of synucleinopathies (12,20–22).

In recent years, diagnostic assays based on the amplification of misfolded proteins, originally developed to detect the self-propagating scrapie isoform of prion protein (PrPSc) in prion diseases, have been successfully applied to amplify and detect misfolded α-syn present in the cerebrospinal fluid (CSF) of patients suffering from synucleinopathies (23–25). These techniques, also named seed amplification assays (SAAs), include Real­Time Quaking Induced Conversion (RT­QuIC) and Protein Misfolding Cyclic Amplification (PMCA). They hold great promise for differentiating patients with synucleinopathies from other diseases, even at a prodromal or early stage. However, distinguishing between the different types of synucleinopathies is still a major challenge. The different kinetic profiles observed during SAAs have suggested that different α-syn strains are amplified in different pathologies (21,26). This has been confirmed by the analysis of patient-derived α-syn fibrils obtained after SAAs by transmission electron microscopy (TEM) and by the observation of specific proteolysis patterns by SDS-PAGE after exposure to Proteinase K (21,20,27). Therefore, analysis of the structure of α-syn fibrils after SAAs may improve the diagnosis of synucleinopathies.

The use of conformational-specific ligands that can differentially bind to different α-syn fibrillar polymorphs would be one way to increase the throughput of such an analysis. Nucleic acid-based ligands, named aptamers, seems particularly promising for this purpose. Aptamers are identified from a large library of 10^15^ different sequences using SELEX (Systematic Evolution of Ligands by Exponential enrichment), which is a molecular evolution process (28,29). The high specificity of aptamers has already been used to detect changes in enzymes conformation (30–32) and to verify the conformational state of therapeutic protein products (33,34). These results lead us to ask whether aptamers can be designed to bind specifically to a particular α-syn fibrillar polymorph. Here, we report the *in vitro* selection of several nuclease resistant 2′Fluoro-Pyrimidines (2′F-Py) RNA aptamers that can specifically bind to an α-syn fibrillar polymorph, called fibrils (or F-type). These aptamers are designed to discriminate this polymorph from another polymorph, called ribbons (or R-type). We also develop a new method, called AptaFOOT-Seq, which uses NGS (Next-Generation Sequencing) to generate different footprints that can identify different polymorphs by measuring the binding of a mixture of aptamers. This method can distinguish different α-syn fibrillar polymorphs derived from patients with PD, DLB or MSA, providing a new way to improve synucleinopathies diagnosis.

## Materials and methods

### Reagents

Unless otherwise stated, the chemical reagents were purchased from Sigma-Aldrich, the molecular biology reagents were purchased from Thermo Fisher Scientific and the oligonucleotides were purchased from Eurogentec. To reduce non-specific binding of α-syn fibrils to plastic, proteins were always manipulated in Eppendorf Protein LoBind tubes.

### Preparation of α-synuclein fibrils.

Two α-syn fibrillar polymorphs, called fibrils and ribbons (or F-type and R-type, respectively) were obtained as previously described (14). Basically, the F-type polymorph was obtained by incubating 300 μM αSyn in 50 mM Tris–HCl, pH 7.5, 150 mM KCl. The R-type polymorph was obtained by dialysing 300μM αSyn against 1000 volume of 5mM Tris–HCl, pH 7.5 for 16 h at 4°C. The assembly of F- and R-type polymorphs was achieved at 37°C, under continuous shaking in an Eppendorf Thermomixer set at 600 r.p.m for 7 days. Patient derived αSYN assemblies were obtained after protein misfolding cyclic amplification (PMCA) assay from frozen brain tissue homogenates of patients suffering from PD, DLB and MSA as previously described (20). The tissues were obtained at autopsy from four to five patients per condition through the UK Brain Bank (Imperial College London, UK). The clinicopathological description of the 19 patients is summarized in (20). The quality of the fibrillar assemblies was assessed by transmission electron microscopy and limited proteolysis by proteinase K as previously described (20).

### 2′F-py RNA pool synthesis

A 2′F-Py RNA pool was synthesized and purified as previously described (35). Briefly, a single-stranded DNA library (‘5-CCTACACGACGCTCTTCCGATCT(N35)TCAGCCTCAACGGATACTCTCCC-3′) consisting of a 35-nucleotide random region (N35) flanked by two constant regions was chemically synthesized and purified by denaturing polyacrylamide gel electrophoresis. The library was first extended into a double-stranded DNA template using a primer called P72 (‘5-TAATACGACTCACTATAGGGAGTATCCGTTGAGGCTGA-3′), which provides a promoter sequence for T7 RNA polymerase (sequence underlined). The double-stranded DNA template was purified by non-denaturing polyacrylamide gel electrophoresis and 2 nmol (approximately 10^15^ different sequences) were *in vitro* transcribed into 2′F-Py RNA sequences, further purified by denaturing polyacrylamide gel electrophoresis and quantified using a NanoDrop spectrophotometer (Thermo Fisher Scientific).

### Selection of aptamers

**Figure 1.**
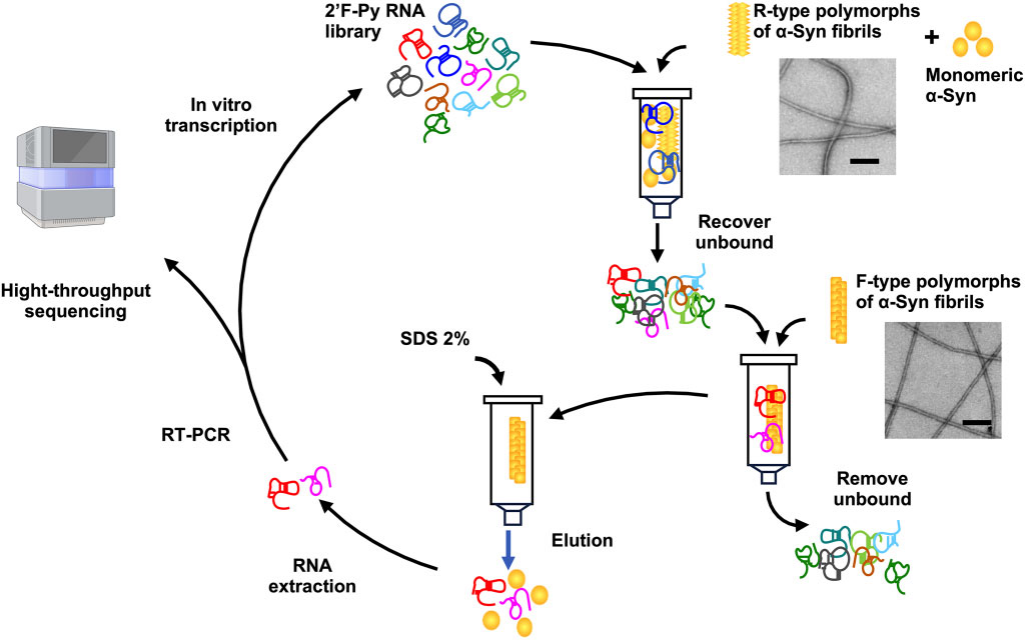
Schematic representation of *in vitro* selection S2 against F-type α-syn fibrillar polymorphs of α-Syn fibrils. The 2′F-Py RNA library is first incubated with α-syn monomers and R-type α-syn fibrillar polymorphs before passing through Sp6 Bio-gel Micro Bio-Spin columns, which retain α-syn. Oligonucleotides that can pass this counter-selection step are then incubated with F-type α-syn fibrillar polymorphs before passing through Sp6 Bio-gel. During this positive selection step, aptamer that can bind to F-type polymorphs are retained by the Bio-gel. After several washes, 2% SDS is added to depolymerize the α-Syn fibrils and elute the aptamers, which are recovered by phenol-chloroform extraction and ethanol precipitation before being amplified by RT-PCR and *in vitro* transcription. After each round of selection, an aliquot of the library is retrieved for NGS sequencing. This strategy was used for SELEX S2, SELEX S1 used the same strategy but without α-syn monomers and R-type α-syn fibrillar polymorphs. Transmission-electron microscopy images after staining with Uranyl acetate illustrate the morphological differences between polymorphs. Scale bar, 200 nm.

Prior to use, the 2′F-Py RNA library was always heated at 85°C for 5 min, cooled on ice for 5 min, and allowed to warm at 37°C until use to disrupt intermolecular interactions between sequences. The selection was performed using Micro Bio-Spin 6 columns (Biorad) which contains Bio-Gel *P*-6 gels. Micro Bio-Spin 6 columns were first equilibrated in the TS selection buffer (10mM HEPES pH 7–7.6, 150 mM NaCl, 5 mM KCl, 1.5mM CaCl_2_ and 1 mM MgCl_2_) by four washes of 500μl TS buffer according to the supplier's instructions. These columns are generally used for gel filtration by centrifugation at 1000g for a few minutes with a molecular weight exclusion limit of 6000 Da. However, we observed that α-syn, either in monomeric form or assembled into fibrils, is retained by the Bio-Gel *P*-6 once equilibrated in TS buffer, whereas 2′F RNA can pass through (Supplementary Figure S1). For the first round of SELEX, around 1.8 nmol of 2′F-Py RNA library was first pass through Micro Bio-Spin 6 columns (centrifugation at 1000g during 4min). This counter-selection step was used to remove any aptamers that can bind to the Bio-Gel *P*-6. The library was then incubated for 1 hour at 37°C with F-type α-syn fibrillar polymorphs (corresponding to approximately 1.8 nmoles of α-syn monomer) in a final volume of 180 μl of TS buffer before being split in 4 and passed through Sp6 Micro Bio-Spin columns (45 μl per column, centrifugation at 1000g during 4min). Unbound aptamers were further removed by two additional washes with 75μl TS buffer per column (centrifugation at 1000g during 2min). Bound aptamers were eluted from the columns by two washes of 150 μl 2% SDS per column (centrifugation at 1000g during 4min), which depolymerized the α-syn fibrils and detached them from the Bio-Gel. 2′F-Py RNA sequences were then recovered by Phenol-chloroform extraction, concentrated by Ethanol precipitation, amplified by RT-PCR using P72 and PiRT (‘5-CCTACACGACGCTCTTCCGATCT-3′) primers and *in vitro* transcribed as described elsewhere (35). After purification by denaturing polyacrylamide gel electrophoresis, the 2′F-Py RNA library was split in order to continue SELEX in parallel using two different protocols (S1 and S2). The S1 protocol used the same counter-selection as the first round. The S2 protocol used a more stringent counter-selection (Figure 1), the library was first incubated simultaneously with R-type polymorphs of α-syn fibrils and α-syn monomers (corresponding to approximately 1.8 nmoles of α-synuclein for both) before passing through the columns to eliminate aptamers that could bind to R-type polymorphs of α-syn fibrils, α-syn monomers or Bio-Gel. Except for this difference in counter-selection, 15 rounds of SELEX were performed identically for S1 and S2. The selection pressure was gradually increased over the rounds by increasing the number of washes (from 2 to 7), decreasing the incubation time (from 1h to 10min), decreasing the amount of F-type α-syn fibrillar polymorphs (from 2 to 0.1 nmol) and decreasing the concentration of the library (from 10 to 1 μM).

### Next-generation sequencing (NGS)

For both SELEX, aliquots of the library from rounds 1 to 15 were analyzed by NGS on a NextSeq 500 instrument (Illumina) as previously described (35,36). Approximately 6–15 million sequencing reads were generated for each round of SELEX. The sequencing analysis workflow is presented in Supplementary Figure S2 and has been previously described (37,36). This is performed by several home-made scripts that were used sequentially to analyze the results and generate the corresponding graphs (Excel and GraphPad Prism). In short, the primer binding sites were removed to recover only the sequences corresponding to the random region. Sequences having a random region of 33–37 nucleotides between primer binding sites were recovered because it is very common for sequences to undergo deletions or insertions of a few bases during SELEX. The frequency of each sequence in each round was then calculated. All sequences with a frequency of at least 0.001% in one round were clustered into families based on a Levenstein distance of 7 (i.e. all sequences with less than seven substitutions, deletions or insertions are grouped into the same family). The frequency of each family was then calculated for each round. In this way, the enrichment of the different families as well as of each sequence within a family can be measured (Supplementary Table S1 and S2, respectively). We observed that three conserved sequence motifs were present in several families. The presence of these motifs has been searched in all families tolerating an editing distance of 1. The percentage of the library and the number of families that contain these motifs were then calculated for each round. Multiple alignment of families that contain one of the conserved motif was performed by MultAlin (38) and the conservation of these motifs was analyzed using MEME (39). Structure prediction of aptamers was performed using Mfold (40).

### Preparation of radiolabeled aptamers

A DNA sequence called ‘SpacerG’ (‘5-TAGGCA{G}TCAC{G}CTGGGGCAATGC-3′, where {G} is a guanine in LNA chemistry to stabilise the double helix, was radiolabelled at the 5′ end with [32P] (3–6 MBq/pmol) using a T4 kinase as described previously (37). For the synthesis of 2′F-Py RNA candidate aptamers, chemically synthesised ssDNA templates were amplified by PCR using P72 and PiRT-G (‘5- TAGGCAGTCACGCTGGGGCAATGCCCTACACGCTCTTCCGATCT-3′) primers prior to *in vitro* transcription and purification as described above. Through the use of the extended reverse primer (PiRT-G), a sequence complementary to the SpacerG sequence was added to the 3′ end of each 2′F-Py RNA aptamer candidate. Extended aptamers and [32P] radiolabelled spacerG were then annealed in TS buffer by heating the samples to 85°C for 5 min and cooling to room temperature for 15 min. Hybridisation was confirmed by electrophoresis in a 3% agarose gel. DNA aptamers from the literature were chemically synthesised and purified by Eurogentec. They were then directly radiolabelled with [32P] using the same protocol described for the radiolabelling of SpacerG. All the sequences which have been tested are provided in Supplementary Table S6.

### Radioactive filter-binding assays on *different* α-syn *fibrillar polymorphs*

Filter binding assays were performed using a Whatman Minifold-1 96-well apparatus (GE Healthcare—10 447 900) and a vacuum pump. Nitrocellulose membranes (HAWP00010—0.45 μm, Millipore) pre-treated with KOH according to protocole described in (41) and Hybond N + membranes (GE Healthcare—RPN203B) were equilibrated in TS buffer for 10 min prior to experiments. For screening of candidate aptamers, 10 nM of each sequence hybridised with [32P] radiolabelled SpacerG was incubated for 30 min at 37°C with different polymorphs of α-syn (corresponding to approximately 1μM of α-synuclein monomers). Incubation was performed in 90 μl TS buffer supplemented with 1.7 ng/μl ssDNA and 0.1% IGEPAL detergent (to reduce non-specific binding of aptamers to fibrils and to reduce non-specific retention of fibrils on plastic, respectively). The filtration apparatus was assembled with a nitrocellulose membrane at the top and a Hybond N+ membrane at the bottom. Initially, 100 μl of TS buffer was loaded and removed using the vacuum pump. The binding mixtures were then added and passed through membranes by the vacuum pump. Unbound aptamers were removed by washing twice with 75 μl TS buffer. Bound and unbound aptamers were then quantified after several hours exposure of MultiSensitive Phosphor Screens to nitrocellulose and Hybond N+ membranes, respectively, using a Cyclone Plus Storage Phosphor System (PerkinElmer). Candidate binding was compared with that of a scramble sequence of the same size and chemistry. Screening was performed in three fully independent experiments, each with three technical replicates.

In order to calculate a *K*_d_, the same experiment was performed with different concentration of radioactive aptamers or scrambled sequences reducing the concentration of protein to 250 nM. The apparent *K*_d_ values were determined by fitting binding curves using the model ‘One site—Fit total and nonspecific binding’ from GraphPad Prism 10 considering the binding of the scramble sequence as non-specific binding. Apparent *K*_d_ of aptamers for different α-syn fibrillar polymorphs were calculated from completely independent experiments (in triplicate and duplicate for 2′F-Py RNA and DNA aptamers, respectively). The *K*_d_ was only calculated if the binding curve was at least twice as high as that of the scrambled sequence, otherwise we concluded that there was no binding.

### aptaFOOT-Seq assays

An equimolar mixture of 14 aptamers (F0, F0AC, F0 F1, F2, F3, F4, F5, F15, F20, F30, F62, F73, F124 and F164) and a scrambled sequence was prepared in TS Buffer, aliquoted and stored at -20°C until use. F0AC corresponds to a variant of F0 with two mutations (052C > A/053A > C). All sequences were extended in 3′ with a sequence that can hybridize spacer G sequence, as previously described. The mixture was then incubated in protein Lowbind tubes (Eppendorf) in a final volume of 10μl with or without α-syn fibrillar polymorphs in TS buffer supplemented with 24 ng/μl ssDNA and 0.1% IGEPAL detergent. The condition without α-syn fibrillar polymorphs is used in every aptaFOOT-Seq assays to to verify that there is no RT-PCR bias or cross-contamination between samples. The final concentration of each aptamer was 4.5 μM, and, except when specify, the concentration of α-syn was 250 nM (3.75 ng/μl of protein). The mixture was incubated at 37°C during 20 min then 40μl of TS buffer containing 0.1% IGEPAL detergent is added just before loading on Sp6 Micro Bio-Spin columns equilibrated in the TS selection buffer as previously described. Unbound oligonucleotides are removed by centrifugation at 1000g during 1min. Unless otherwise stated, the column is washed twice with 100 μl of TS buffer (centrifugation at 1000g during 1min). The bound sequences are eluted by 200μl of SDS 2% (centrifugation at 1000g during 4min) before being purified by phenol/chloroform extraction and ethanol precipitation. Alternatively, bound sequences are eluted by 50μl of RAV1 buffer before extraction using a Kit from Macherey-Nagel (NucleoSpin 96 Virus Core). Next, the RNA is reverse transcribed into cDNA, which is sequenced by NGS so as to have around 100 000 reads per assay. A shema of the protocol is provided in Supplementary Figure S6. For each aptamer a, a normalized enrichment ratio (RN) between every condition x and 0 (starting mixture) is calculated using the formula:


\begin{equation*}R{N_a} = \frac{{{r_{a,x}} - \frac{1}{n}}}{{\frac{1}{n}}}\end{equation*}

where *n* is the number of aptamer in the mixture (15 in our case)


\begin{equation*}{r_{a,x}} = \frac{{r_{a,x/0}}}{\sum_{a \in A}{r_{a,x/0}}}\end{equation*}

where *A* = {F0,F0AC,F1,F2,F3,F4,F5,F15,F20,F30,F62,F73,F124,F164,Scr} and


\begin{equation*}{r_{a,x/0}} = \frac{{{f_{a,C = x}}}}{{{f_{a,C = 0}}}}\end{equation*}

where *f*_*a*,*C*=*x*_ is the frequency of aptamer *a* in the mixture at condition *x* and *f*_*a*,*C*=0_ is the frequency of aptamer *a* in the starting mixture. The Sum of all RNs = 0 (If the frequency of one aptamer in the mixture increases, the frequency of at least one other aptamer must decrease). A step-by-step example of the calculations is given in Supplementary Figure S7. For each aptaFOOT-Seq assay, three technical replicates were performed. The footprint was generated by taking the median of the RNs for each aptamer. Hierarchical clustering based on Pearson correlation was performed using TreeView3 version (beta 1) (42).

## Results

### *Selection of* 2′F-py RNA *aptamers against F-type* α-syn *fibrils*

**Figure 2.**
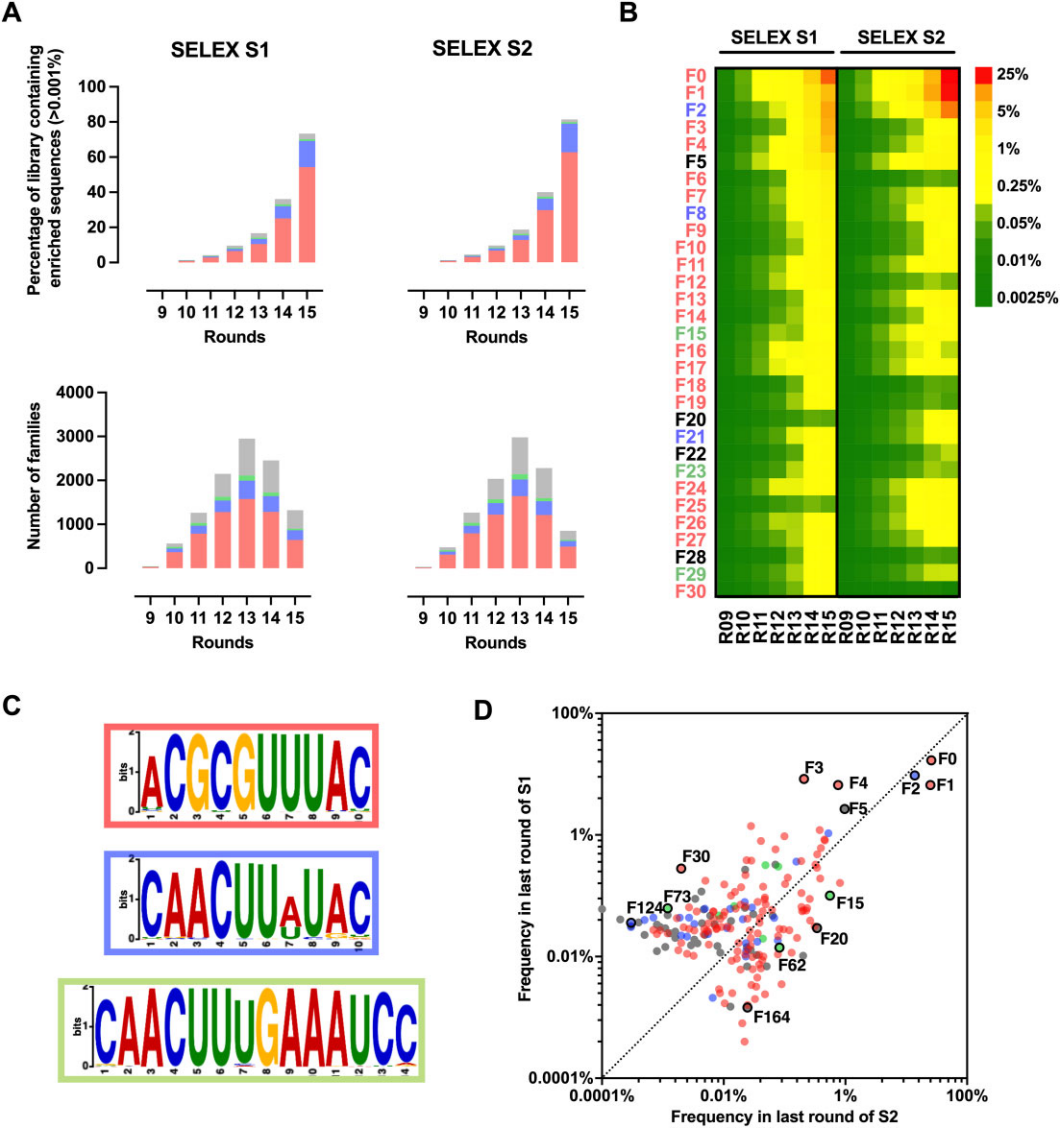
Next Generation sequencing analysis of SELEX S1 and S2. Color code is used throughout the figure. Families containing the motifs CGCGUUUAC, AACUUAUA or CAACUUUGAAAUCC are highlighted in red, blue and green respectively. (**A**) Top panel represent the fraction of the library (in %) corresponding to sequences whose frequency was higher than 0.001% in at least one round of SELEX S1 or S2. These sequences were then clustered into families based on an edit distance of 7. The lower panel A shows the evolution of the number of families during rounds. (**B**) heat map presenting the evolution of the frequencies for the 31 most enriched families. (**C**) Conservation of the three motifs that are common to a large number of families analyzed using MEME. (**D**) Correlation between frequencies at the last rounds of SELEX S1 and S2 for the 200 most abundant families. The dots circled in black correspond to the families that were selected for affinity evaluation in Figure 3.

We used two distinct *de novo* generated α-syn fibrillar polymorphs termed ‘Fibrils’ and ‘Ribbons’, or F-type and R-type, respectively, to select conformational aptamers. These fibrillar polymorphs exhibit distinct structures, nanomechanical properties, shapes in the electron microscope and surfaces exposed to the solvent (14–17). Furthermore, these polymorphs demonstrated different *in vitro* and *in vivo* seeding and propagation properties (12,18,19). Two SELEX strategies were used in parallel to isolate aptamers able to bind specifically to F-type α-syn fibrillar polymorphs. The first (SELEX S1) used counter-selection against the selection support (Bio-gel P 6), whereas the second (SELEX S2) used a more stringent counter-selection against the selection support as well as the R-type α-syn fibrillar polymorphs and the monomeric form of the protein (Figure 1). 15 rounds of selection were carried out for both selections and the stringency was varied to progressively increase the selection pressure. The enrichment of certain sequences in the library was analyzed by NGS. Approximately 10 million sequences were analyzed per round. Of the 85 million unique sequences in the analysis, 9 021 sequences had a frequency in the library that was greater than 0.001% in at least one round. These sequences were retrieved and grouped into families (Supplementary Table S1). We observed that most families contain similar sequences with no more than three substitutions, insertions, or deletions (Supplementary Table S2). The frequencies of many families increase over the two SELEXs. We observed that the sum of the frequencies of these families increased exponentially in both SELEX experiments, representing up to 70% of the library in the final rounds (Figure 2A top panels and Supplementary Figure S3). Some families were amplified more efficiently than others, resulting in a decrease in family diversity from round 13 (Figure 2A, bottom panels). Many families were enriched in the same way in both SELEXs, despite the fact that they originated from different selection paths (Figure 2B, D and Supplementary Table S1). For example, the most abundant family (F0) represented up to 17 and 26% of the library in the last round of SELEX S1 and S2, respectively. However, some families were much more enriched in one SELEX than the other, for example F15, F20 or F30 (Figure 2B and D). Three motifs conserved across many families were identified by aligning the 200 most abundant families, motif 1: ACGCGUUUAC, motif 2: CAACUUAUAC and motif 3: CAACCUUUGAAAUCC. The presence of these three motifs was searched in all families and their conservation among the families was analyzed using the MEME suite software (Figure 2C, Supplementary Table S3–S5). None of the motifs appear to be more enriched than the others during the SELEX rounds, although motif 1 appears more abundant than motif 2, which in turn is much more abundant than motif 3 (Figure 2A). We therefore decided to assess the affinity of 13 families including 5 that were predominantly enriched in both SELEX (F0, F1, F2, F4 and F5) and 4 families that were predominantly enriched in either SELEX S1 (F3, F30, F73 and F124) or SELEX S2 (F15, F20, F62 and F164), ensuring that several of these families contained one of the three motifs we identified (Figure 2D).

### Screening of 2′F-py RNA candidates against different α-syn fibrillar polymorphs

**Figure 3.**
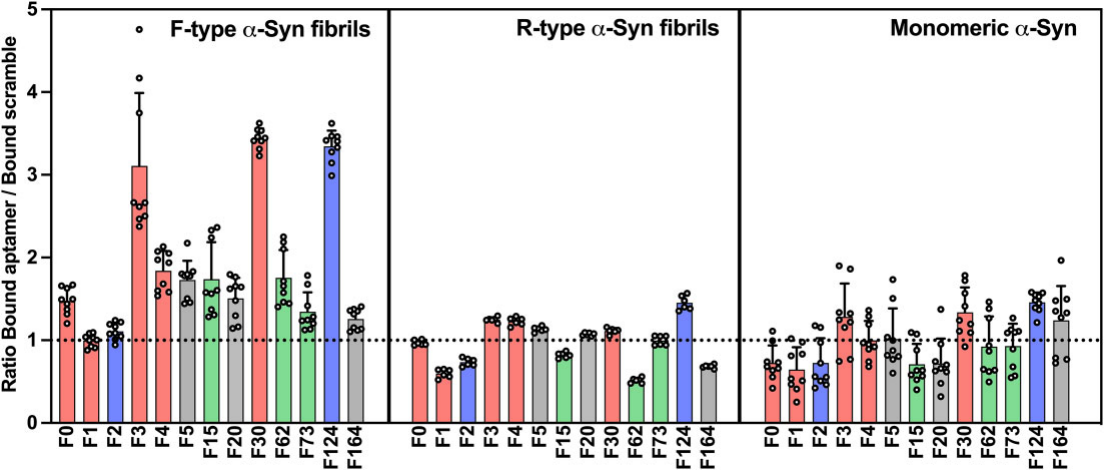
Screening of aptamer candidates against different α-Syn conformers. Screening assays of aptamer family candidates against F- or R-type α-Syn fibrillar polymorphs and monomeric form of α-Syn. 10nM of aptamer candidates previously hybridized with a ^32^P radiolabeled SpacerG oligonucleotide were incubated with 1 μM of each conformation of α-Syn before being filtered through mixed cellulose esters (MCE) membrane. After several washes, the amount of aptamer bound to the protein and thus retained on the membrane was compared to the amount bound by a scramble sequence. The same color code as in Figure 2 is used and highlight families that contain one of the three conserved motifs.

Thirteen aptamer candidates were synthesized with an extended 23 nucleotide end before hybridization to a radiolabeled oligonucleotide. This complex was incubated with either F-type or R-type α-syn fibrillar polymorphs or the monomeric form of the protein. Then, the protein bound fraction was quantified after filtration through nitrocellulose membranes. The same experience was performed using a scrambled oligonucleotide sequence of the same size and the same 2′F-Py RNA chemistry. These membrane retention assays were performed in a series of three independent experiments, each with 3 technical replicates. None of the candidates appear to have a higher affinity than the scrambled sequence for either the R-type fibrillar polymorphs or the monomeric form of the protein. In contrast, several candidates had higher binding to the F-type α-syn polymorph compared to the scrambled sequence, in particular F3, F30 and F124, which exhibit at least 3-fold higher binding capacity (Figure 3). Notably, these three aptamers were more enriched in SELEX S1 than in SELEX S2, with at least 40 times higher frequencies in the libraries of the last round of SELEX S1 compared to SELEX S2 (Figure 2D and Supplementary Table S1). F3 and F30 contain motif 1 and F124 motif 2, but the presence of this motif alone cannot explain aptamer binding, since other families containing the same motifs do not bind to F-type α-syn polymorph (for example F1 and F2). Structure prediction of these aptamers indicated that F3 and F30 may adopt a similar asymmetric internal-loop structure, whereas F124 is predicted to adopt a different structure (Supplementary Figure S4). We therefore decided to limit our study to F30 and F124 due to the structural similarities of F3 and F30.

### *Affinity and specificity of 2′F-py RNA aptamers compared to* DNA aptamers previously described in the literature

**Figure 4.**
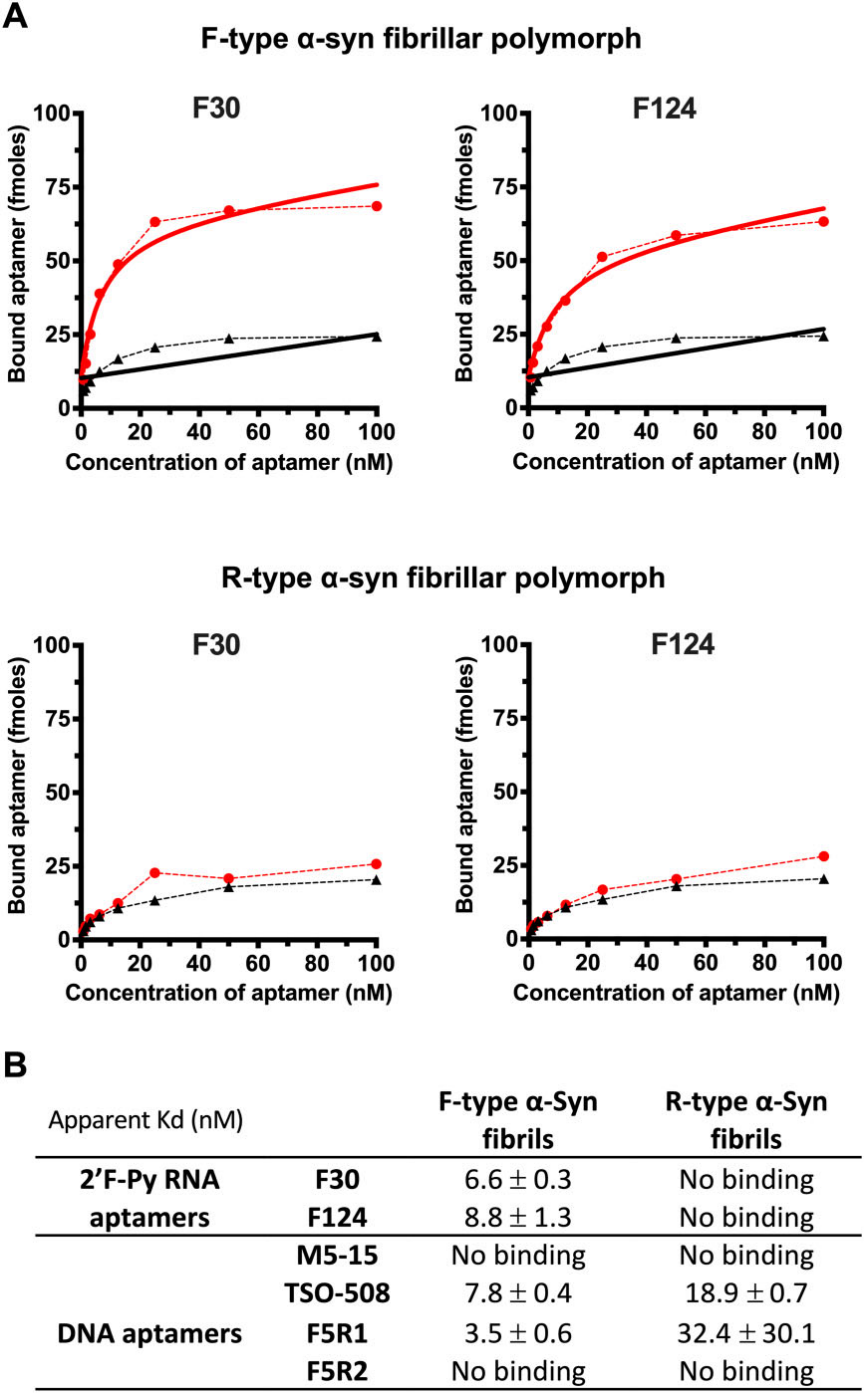
Binding of F30 and F124 2′F-Py RNA aptamers on different F-type and R-type α-Syn fibrillar polymorphs compared to DNA aptamers previously described in the literature. (**A**) example of binding curves of F30 and F124 against F-type (upper panel) and R-type (lower panel) α-Syn fibrillar polymorphs. The binding of aptamers are in red while the binding of a scramble sequence was used to evaluate the nonspecific binding (black dotted curves). The binding curves were fit using the model ‘One site - Fit total and nonspecific binding’ from GraphPad Prism 9 to calculate the apparent *K*_d_ of the interaction. (**B**) Apparent *K*_d_s of 2′F-Py RNA and DNA aptamers on different F-type and R-type α-Syn fibrillar polymorphs. The values presented in the table represent the average of the apparent *K*_d_s and their standard deviation calculated from completely independent experiments (in triplicate and duplicate for 2′F-Py RNA and DNA aptamers, respectively).

Binding of F30 and F124 to F-type and R-type α-syn fibrillar polymorphs at increasing aptamer concentrations (0.8 to100nM) was assessed as described above (Figure 4A). The aptamers could only bind to F-type α-syn fibrillar polymorph, which confirm their specificity, and their apparent dissociation constants (*K*_d_) was 6.6 ± 0.3 and 8.8 ± 1.3 nM for F30 and F124, respectively (Figure 4B). We also decided to compare the affinity and specificity of these 2′F-Py RNA aptamers with four DNA aptamers previously described in the literature as ligands of the monomeric and oligomeric forms of α-syn: the aptamers M5-15 and T-SO508 selected by Tsukakoshi et al. (43,44) and F5R1 and F5R2 selected by Zheng *et al.* (45). From the binding curves we obtained, we could show binding of the two DNA aptamers, TSO-508 and F5R1, to α-syn fibrils under our experimental condition with an apparent Kd of around 10 nM (Figure 4B and Supplementary Figure S5). However, in contrast to the F30 and F124 aptamers we describe here, DNA aptamers bind both F-type and R-type α-syn fibrillar polymorphs, although with higher affinity (2–10-fold) for the F-type polymorph.

### Development and optimisation of the AptaFOOT-Seq method to discriminate F- from R-types α-syn fibrillar polymorphs.

**Figure 5.**
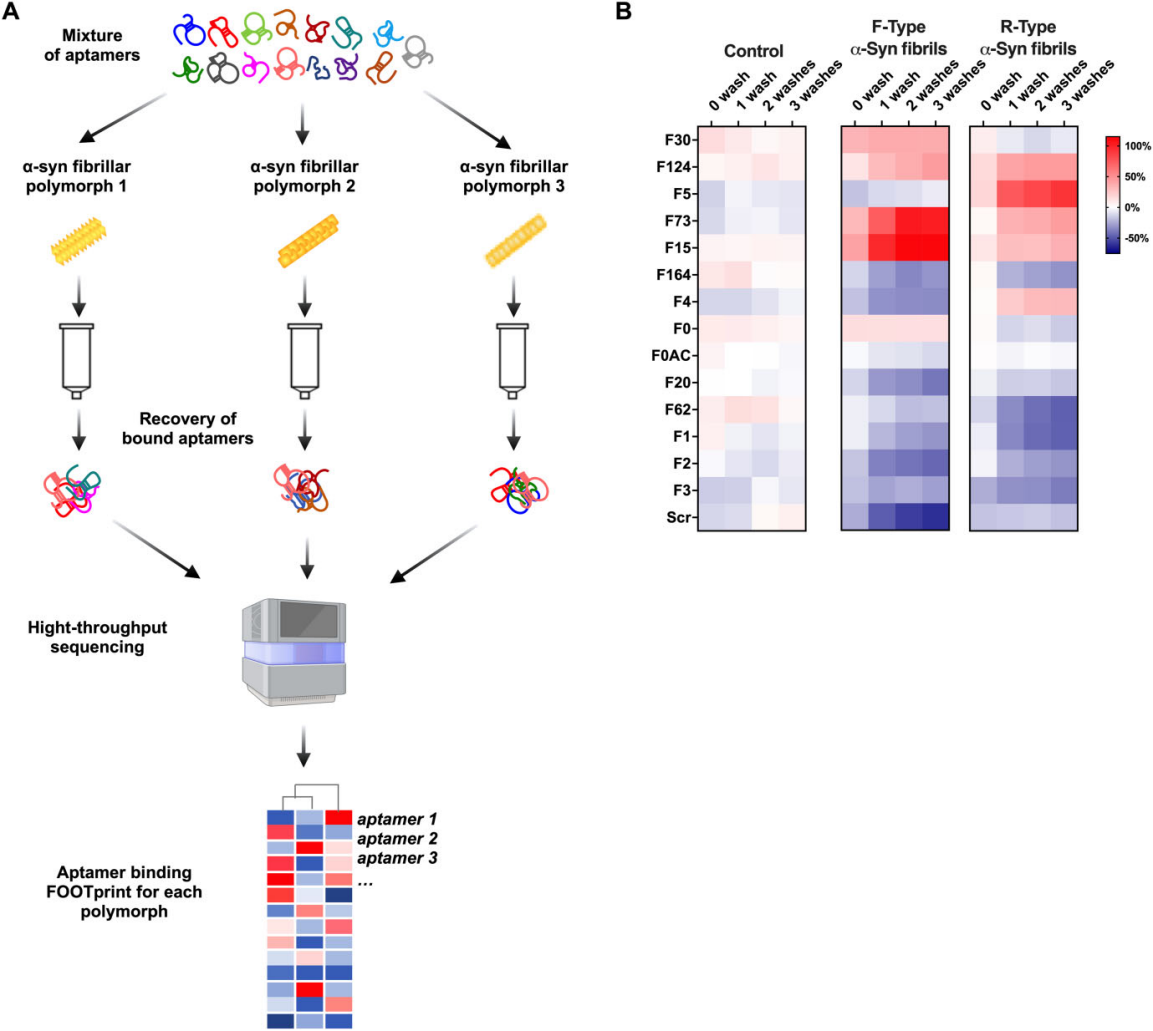
Optimization of the AptaFoot-Seq method. (**A**) Schematic representation of an aptaFOOT-Seq experiment. A define mixture of aptamers and a control scramble sequence is first incubated with different α-Syn fibrillar polymorphs before being pass through Sp6 Bio-gel Micro Bio-Spin columns. Unbound aptamers are removed by several washes. Then, aptamers that bind to the polymorphs are eluted and amplified by RT-PCR with specific index sequence for each conformer. These libraries can then be pooled and sequenced. After demultiplexing, the fold enrichment of each aptamer compared to the starting library can be calculated for each conformer. This differential binding can serve as a FOOTprint that can be used to discriminate different polymorphs. (**B**) FOOTPRINT from experiment without protein and with F-type or R-type α-Syn fibrillar polymorphs after an increase in the number of washes. The median frequency of each sequence was calculated from a triplicate for all footprints.

**Figure 6.**
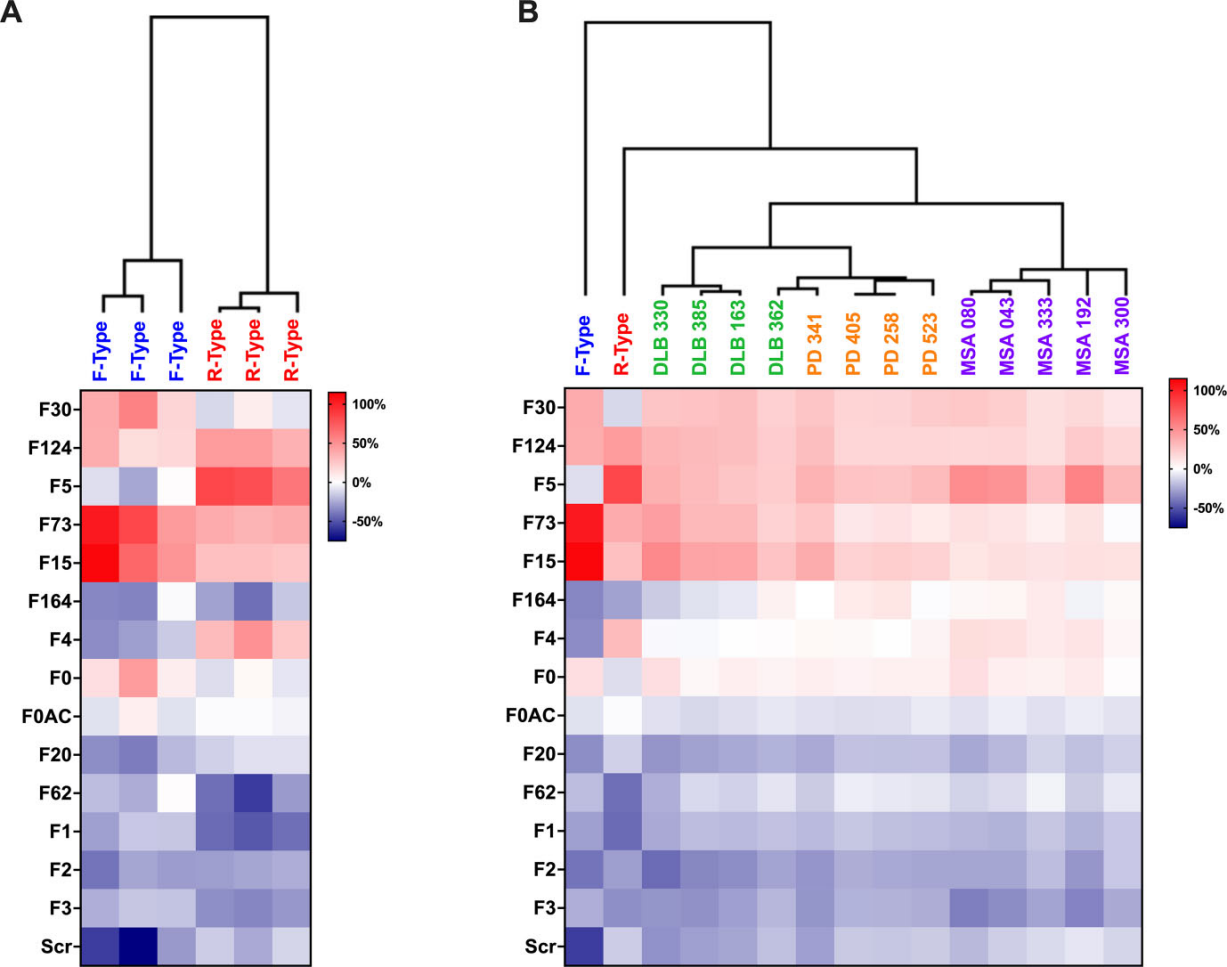
Discrimination between F-type and R-type α-Syn fibrillar polymorphs using AptaFOOT-Seq and evaluation on α-Syn fibrils from patients with different synucleopathies. (**A**) The the AptaFOOT-Seq method was evaluated by three fully independent experiments against recombinant F-type or R-type α-Syn fibrillar polymorphs. The median frequency of each sequence was calculated from a triplicate for all footprints. The FOOTprints were used to perform hierarchical clustering based on Pearson correlation. This clustering allows a tree to be drawn that can cluster fibrils of different origins based on the calculated distance matrix between the FOOTprint data. The length of the branches corresponds to the calculated distance between the FOOTprints. This distance reflects the degree of similarity between the conditions: shorter distances indicate greater similarity, while longer distances indicate greater dissimilarity. (**B**) Footprints obtained with α-Syn fibrils amplified by PMCA from PD, MSA and DLB patients and with F-type or R-type α-Syn fibrillar polymorphs. Hierarchical clustering was performed as in (A).

Both F30 and F124 aptamers can be used to distinguish F and R-type α-syn fibrillar polymorphs. However, the extent to which these polymorphs exhibit structural similarities with those present in patient brains is unknown. In addition, we observed during the SELEX screening that several other aptamers had a slightly higher affinity for F-type α-syn fibrillar polymorphs compared to a control sequence (Figure 3). We therefore decided to develop a method that could rapidly test the affinity of all these aptamers for different α-syn fibrillar polymorphs by harnessing the power of NGS. We call this method ‘aptaFOOT-Seq’ because it provides an aptamer binding footprint using NGS. AptaFOOT-Seq principle is represented in Figure 5A. First, all the tested aptamers are mixed and a control sequence that can be used as a negative control is added. The mixture is then incubated with different α-syn fibrillar polymorph. After several washes, the aptamers bound to each polymorph are recovered and amplified by RT-PCR, adding an index sequence that can be used for multiplex sequencing to analyze in parallel several assays against different polymorphs. Finally, for each aptamer, its enrichment with respect to the starting mixture can be calculated to build a specific ‘footprint’ corresponding to the relative binding of each aptamer to a given polymorph as compared to the others. The aptaFOOT-Seq method was first evaluated with F- and R-type α-syn fibrillar polymorphs, varying the number of washes (Figure 5B). The abundance of each aptamer in the mixture showed no significant change after selection on Sp6 columns in the absence of α-syn fibrillar polymorphs. This shows that no aptamer is retained significantly more than another in the columns and that each sequence is amplified by RT-PCR with similar efficiency and without bias. In contrast, some aptamers were retained to a much greater extent than others in the presence of F- and R-type α-syn fibrillar polymorphs. However, the footprints were a bit surprising. Indeed, while higher retention of F30 and F124 to F-type α-syn fibrillar polymorph was expected, there was a much higher retention of F73 and F15, which exhibit a lower binding propensity when screened individually (Figure 3). Furthermore, we observed a higher retention of several aptamers by R-type α-syn fibrillar polymorph, including the F124 aptamers while no aptamer seems to bind to this polymorph when they were evaluated individually. We suspect that competition or cooperativity between aptamers affects their binding when mixed. But, we realised that the aptaFOOT-Seq method we implemented was able to provide a very different footprint for each polymorph. Furthermore, a single wash of the column with 100μl of buffer was sufficient to obtain footprints that remained constant after two or three washes. Next, we decided to challenge the reproducibility of the aptaFOOT-Seq method repeating the experiment with two washes in three fully independent experiments (Supplementary Figure S8). AptaFoot-Seq results show a linear correlation between experiments, demonstrating the robustness and reliability of the method with *R*^2^ around 0.854 ± 0.078 and 0.943 ± 0.036 for F-type and R-type α-Syn fibrillar polymorphs, respectively. Next, the FOOTprints were classified using a hierarchical clustering based on Pearson correlation. This clustering allows a tree to be drawn that can cluster fibrils based on the calculated distance matrix between the Footprint data. This clustering shown that the footprints can discriminate easily the F-type from the R-type α-syn fibrillar polymorphs (Figure 6A).

### Evaluation of AptaFOOT-Seq on α-syn fibrils derived from patients with different synucleinopathies

The AptaFOOT-Seq method was next used to discriminate α-syn fibrillar polymorphs derived from four to five deceased patients with a clinical and neuropathological diagnosis of PD, DLB or MSA. To this aim, pure α-syn fibrillar polymorphs were derived from crude brain homogenates by a protein misfolding cyclic amplification (PMCA) assay we recently implemented (20). AptaFOOT-Seq analysis reveals that patient-derived α-syn aggregates have a distinct footprint from F- and R-type α-syn fibrillar polymorphs (Figure 6B). A hierarchical clustering based on Pearson correlation reveals that the footprints of the F-type and R-type are far from those of the patients, indicating that the *de novo* assembled α-syn fibrillar polymorphs can be distinguished from those derived from patients. The footprints obtained with patient-derived fibrillar polymorphs appear to some extent further related. Nonetheless, we could discriminate α-syn fibrils derived from different synucleinopathies, except in one case (DLB 362) where the fibrils yield a footprint closer to those derived from PD patients than from those of the other DLB patients.

## Discussion

Here, we identify aptamers that bind differentially to structurally distinct α-syn fibrillar polymorphs. To identify such conformational ligands, we compared two different strategies, a basic SELEX protocol (S1) against a pure α-syn fibrillar polymorph designated F-type and a more stringent protocol (S2) that use counter-selection steps against another fibrillar polymorph we named R-type. Both SELEX strategies amplified mostly the same aptamer families and the most interesting aptamers (F3, F30 and F124) were much more amplified in S1 than in S2. Therefore, counter-selection seems unnecessary and even detrimental in our case to select conformation-specific α-syn fibrillar polymorphs ligands. Two 2′Fluoro-aptamers we selected (F30 and F124) appear to bind specifically the F-type polymorph (e.g. neither recognize the R-type polymorph nor the monomeric form of α-syn). The selectivity we reached contrasts with that of the DNA aptamers described so far in the literature and is certainly due to the use of pure α-syn fibrillar polymorphs in the SELEXs we performed.

Aptamers have been shown to be highly specific for the 3D conformation of their target. This may account for their ability to detect subtle changes in protein conformation. Accordingly, changes in binding kinetics and equilibrium affinity constants of aptamers have already been used to detect structural differences between proteins, for instance between recombinant human erythropoietin products of different origins (34). This has also been used to detect structural differences of the therapeutic antibody (rituximab) upon incubation at 40°C for 72 h or UV exposure (33). For this reason, aptamers have been proposed to replace tedious techniques such as X-ray crystallography or NMR to control the tertiary structure of therapeutic protein batches. However, accurately measuring the affinity of aptamers can be quite laborious and expensive. In addition, it is recognized that patient-derived fibrils may have different conformations to those formed *in vitro* (46,47). Consequently, while our focus was primarily on the characterisation of two aptamers (F30 and F124) that were considered most promising for *in vitro* assembled α-syn fibrillar polymorphs, the uncertainty regarding their efficacy against patient-derived fibrils prompted the development of the AptaFOOT-Seq methodology. This novel strategy is based on measuring the binding of a mixture of aptamers rather than the measuring the precise affinity of individual aptamers.

This method benefits from advances in high-throughput sequencing which has already been widely used to analyse the evolution of aptamer libraries during several rounds of SELEX and to study the effect of a selection pressure (37,48). For example, the evolution of a library during selection in the presence or absence of a target is often used to better identify aptamers (49–51). Dupont and al demonstrated that such approach can identify aptamers against different target variants of the serpin plasminogen activator inhibitor-1 (PAI-1) (32). The same group has also developed a method, named APTASHAPE, that analyze libraries of SELEX against human plasma to discriminate patients with bladder cancer (52). In that case, a pool of 10^15^ 2′FPy RNAs was first pre-selected by four rounds of SELEX against total human plasma protein from patients suffering from bladder cancer. Then, this preselected library containing a huge number of different sequences with a high diversity of frequencies was incubated with human plasma protein from healthy subject or from bladder cancer patients. Studying the differential enrichment of 1000 most abundant sequences by NGS, allowed differentiating healthy controls from patients diagnosed with bladder cancer. Similar approaches were used to discriminate between blood serum from transgenic Alzheimer's disease model mice from healthy counterparts (53) or to distinguish exosomes from healthy subject and patient with breast cancer (54). Although these studies have demonstrated that SELEX-derived libraries can be used directly, we believe that the aptaFOOT-Seq method has several advantages for application in clinical diagnostics. First, it uses a mixture of a small set of aptamers with a defined frequency, rather than using a library from a SELEX round containing a large number of aptamers with high frequency heterogeneity, some of which are of low abundance. As a result, the composition of the aptamer mixture is easier to generate in a reproducible and controlled manner. In addition, previous studies required a relatively high sequencing depth (at least several million reads per sample) with the associated costs, whereas the aptaFOOT-Seq method requires only a few thousand reads.

This study also sheds new insight into the SELEX strategy. Indeed, it has been reported repeatedly that the most enriched sequences in a SELEX are not always the best ligands when tested individually. Although not demonstrated yet, this has been attributed to the amplification of parasite sequences that have the ability to amplify better during PCR. Our results suggest another explanation. Indeed, the aptamers F73 and F15 exhibited the highest retention by the F-type α-syn fibrillar polymorph in the aptaFOOT-seq method we performed using aptamers mixture, while aptamers F30 and F124 showed at least 2 times higher retention when the aptamers were evaluated individually (see Figure 3 versus Figure 5). Similarly, several aptamers showed higher retention by AptaFOOT-Seq to R-type polymorphs, whereas they did not appear to bind when tested individually. When using a mixture of aptamers as in AptaFOOT-Seq or during SELEX, it is conceivable that aptamers competing for the same interaction site may be less retained than those that are not in competition (Supplementary Figure S9B). In addition, multiple aptamers may interact with each other to form a complex that can interact with the target (Supplementary Figure S9C). Such a complex of several aptamers has been observed in the selection of aptamers against phospholipid bilayers (55). Allostery may also be involved, requiring the interaction of a first aptamer on a target, leading to a conformational change in this target, allowing the binding of a second aptamer (Supplementary Figure S9D). All of these and other events can affect the binding of an aptamer in a mixture. We performed an experiment to verify this hypothesis (Supplementary Figure S10). Each aptamer and the scramble sequence were first independently bound to F- or R-type α-syn fibrillar polymorphs under identical conditions employed for AptaFOOT-Seq. Then, the retained amount for each aptamer was individually reverse transcribed into cDNA before being combined for a single PCR and subsequent sequencing. Consequently, relative binding profiles similar to those generated by AptaFOOT-Seq were reconstructed. In this case, the binding profiles reflect the cumulative binding of individual aptamers assessed separately. We observed a high retention of F5 by both α-syn fibrillar polymorphs, inconsistent with previous radioactive assays (Figure 3). This discrepancy may be due to the slightly different experimental conditions (100nM aptamer and filtration on nitrocellulose as opposed to 5nM aptamer and filtration on Sp6 columns). Apart from F5, the relative binding patterns of other aptamers remained consistent with prior radioactive assays. Notably, the relative binding was much higher for F30 and F124 and lower for F73 and F15 to F-type α-syn fibrillar polymorphs when tested alone compared to AptaFOOT-seq results. For the R-type α-syn fibrillar polymorphs, the relative binding was also lower for F124 and F15. Thus, the evident disparities between these profiles and those obtained via AptaFOOT-Seq confirm that aptamers’ relative binding in a mixture does not merely reflect the sum of their individual bindings assessed separately. The aptaFOOT-Seq method we designed discriminates de novo-assembled and patient-derived α-syn fibrillar polymorphs. We have previously shown that amplification of de novo assembled F- and R-type α-syn polymorphs under exactly the same experimental conditions used for pathogenic α-syn in patients brain yield fibrils that retain the transmission electron microscopy characteristics and limited proteolytic patterns of the original fibrils (46). The latter fibrils are structurally distinct from those we obtain from PD and MSA patients (47), suggesting that the method yields distinct polymorphs with different input seeds. It is worth noting that this amplification process occurs under physiological conditions, in the absence of detergents commonly used to purify pathogenic α-syn aggregates from patient brains, and in the absence of ligands, such as thioflavin T (ThT), which may bind preferentially bind to a subset of fibrillar α-syn polymorphs and thus bias aggregation towards the polymorphs with the highest binding capacity. Therefore, while we cannot definitively claim that the α-syn fibril polymorphs amplified by PMCA perfectly reflect those found in patients, our investigations confirm that they have structural differences that can be exploited to distinguish patients with different synucleinopathies. Accordingly, aptaFOOT-Seq discriminates α-syn fibrils from patients suffering from PD, DLB and MSA. This is consistent with the fact that these fibrils have been shown to induce different neurotoxicity after intracerebral inoculation into the rat substantia nigra, suggesting that they may have distinct conformations. As a result, we believe that aptaFOOT-Seq can detect even small differences in pathogenic α-syn aggregates that are difficult to detect by other methods. This result is significant because different alpha-Syn fibrillar polymorphs may be associated with different clinical manifestations and/or disease progression rates. Understanding this diversity is therefore crucial for advancing our knowledge of the disease mechanisms and for developing targeted therapies. Thanks to NGS, the aptaFOOT-Seq method is easier to perform than measuring the affinity of several aptamers individually. We believe that the aptaFOOT-Seq method is compatible with large-scale population diagnostics. First, the test is very simple and requires few steps that can be easily automated and parallelized (incubation with target, filtration, washing, elution, RNA extraction, RT-PCR, NGS). Parallel measurements can also be performed at minimal cost by using sequencing as a readout. We currently use between 50 000 and 100 000 reads per FOOTprint, which can be obtained for pennies on some sequencers.

Although encouraging, the analysis we performed needs to be validated in a larger number of patients to better determine its accuracy and its sensitivity, as well as to train machine learning algorithms to identify patterns and correlations to improve our diagnostic power. We found that α-syn fibrils from one patient with DLB (DLB 362) provide a FOOTprint closer to those of fibrillar polymorphs from PD patients than from those of the other DLB patients. This either reflects FOOTprint limitations or misdiagnosis. While the aptaFOOT-Seq method is particularly effective in discriminating between F-type and R-type α-syn fibrillar polymorphs, we also observed that the FOOTprint differences between polymorphs amplified from patients are less pronounced. This may be due to the fact that the patient-derived α-syn may exhibit structural similarities. Another explanation could be that the aptamers were selected against F-type fibrillar polymorphs that exhibit a structure different from that of the patients. It is plausible that an APTASHAPE-type approach, starting with a round of SELEX followed by a round of *in vitro* selection of patient-derived fibrils, we could identify other aptamers that could better discriminate fibrils from different synucleinopathies. The polymorphs we used throughout this study were amplified from post-mortem brain tissues. Assessing the recognition patterns of α-syn fibrils amplified from cerebrospinal fluid or even blood samples holds potential from a clinical stand point.

In conclusion, we envisage that aptaFOOT-Seq could be used in a variety of applications where the detection of misfolded or abnormal protein conformations is critical, such as diagnostics, quality control of therapeutic proteins or environmental monitoring. In essence, this method measures the behaviour of a controlled *in vitro* ecosystem of aptamers, where cooperation and competition between individuals can provide a higher level of information.

## Supplementary Material

gkae544_Supplemental_Files

## Data Availability

All fastq files of SELEX are available in the European Nucleotide Archive (ENA) at https://www.ebi.ac.uk/ena/browser/home under accession code PRJEB70964.

## Notes

Present address: Alix Bouvier-Müller, Institut Pasteur, Department of Structural Biology and Chemistry, Laboratory for Bioorganic Chemistry of Nucleic Acids, CNRS UMR3523, Paris, France.

Present address: Alexis Fenyi, AC Immune SA, Building B, EPFL innovation Park, CH-1015 Lausanne, Switzerland.
