# Supplementary material for: Aptamer binding footprints discriminate α-synuclein fibrillar polymorphs from different synucleinopathies
Source: Nucleic Acids Res. 2024 Jun 25;52(14):8072–85. doi: 10.1093/nar/gkae544 (PMC11317169; doi:10.1093/nar/gkae544)
Supplement: gkae544_Supplemental_Files [file gkae544_supplemental_files.zip › Supplementary_Figures_vrevised.pdf]

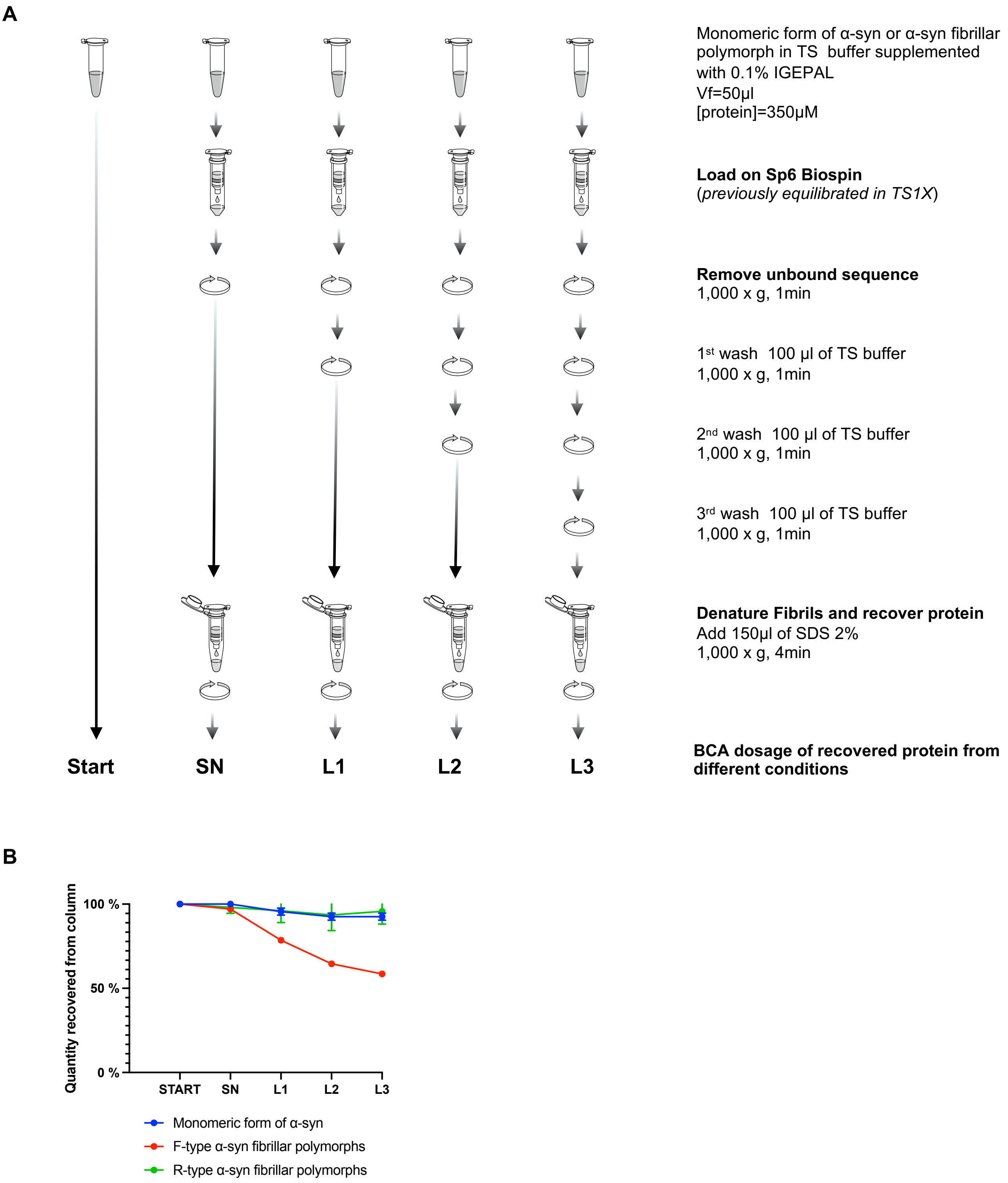

Supplementary Figure S1: Retention of different forms of α-syn on Bio-Gel P-6. The retention of α-syn on Bio-Gel P-6, either in monomeric form or assembled into F-type or R-type fibrillar polymorphs, has been measured by BCA dosage. (**A**) Shema of the protocol. (**B**) Quantification of retention after several washes by BCA. The monomeric form or R-type fibrillar polymorphs are retained at almost 100% even after several washes. The F-type fibrillar polymorphs show a gradual release from the column during the washes, but, more than 50% remain on the column even after three consecutive washes, which was considered sufficient for SELEX.

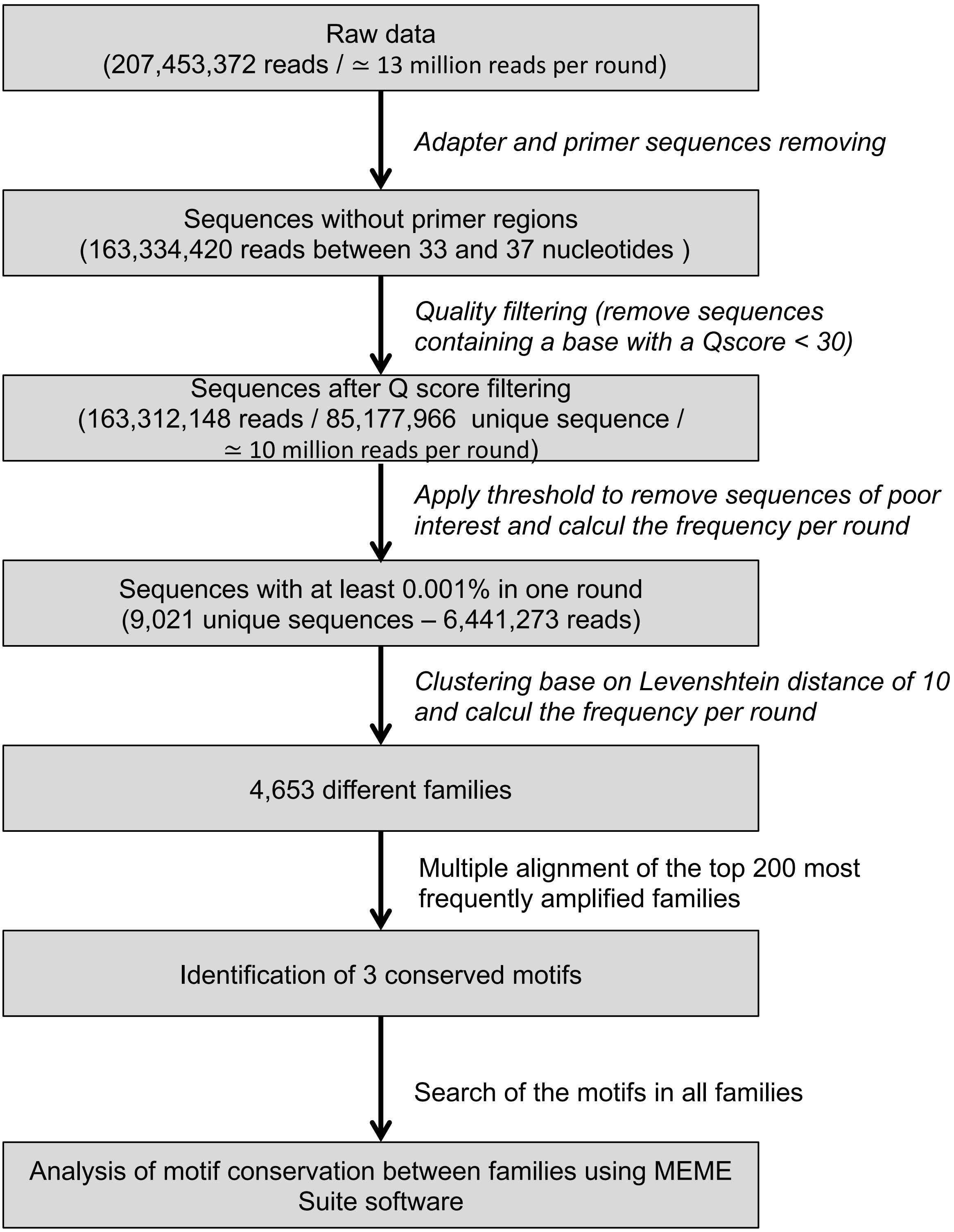

Supplementary Figure S2: Sequencing analysis workflow

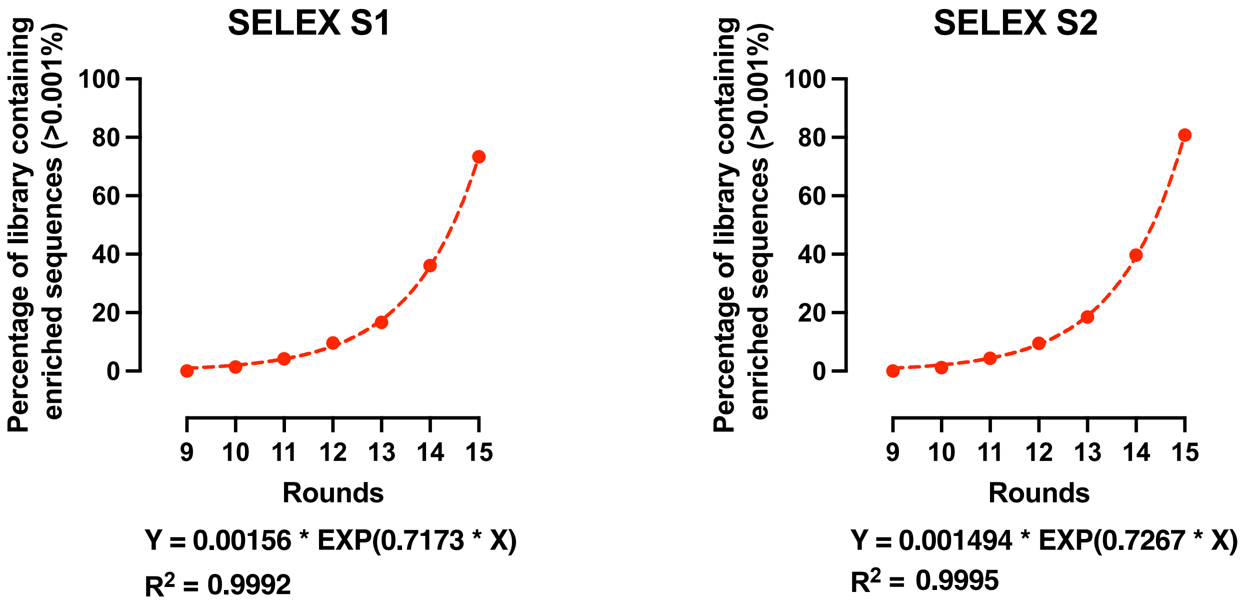

Supplementary Figure S3: Exponential enrichment of sequences with a frequency greater than 0.001%. 9,021 sequences with a frequency in the library that was greater than 0.001% in at least one round were recovered and their frequency in each round was measured. The sum of the frequencies of these sequences in each round of SELEX was plotted on the graph. The growth of these sums was fitted (dotted line) using the “Exponential growth equation" model from GraphPad Prism 10. The equations of the fits and the R^2 are given below each graph.

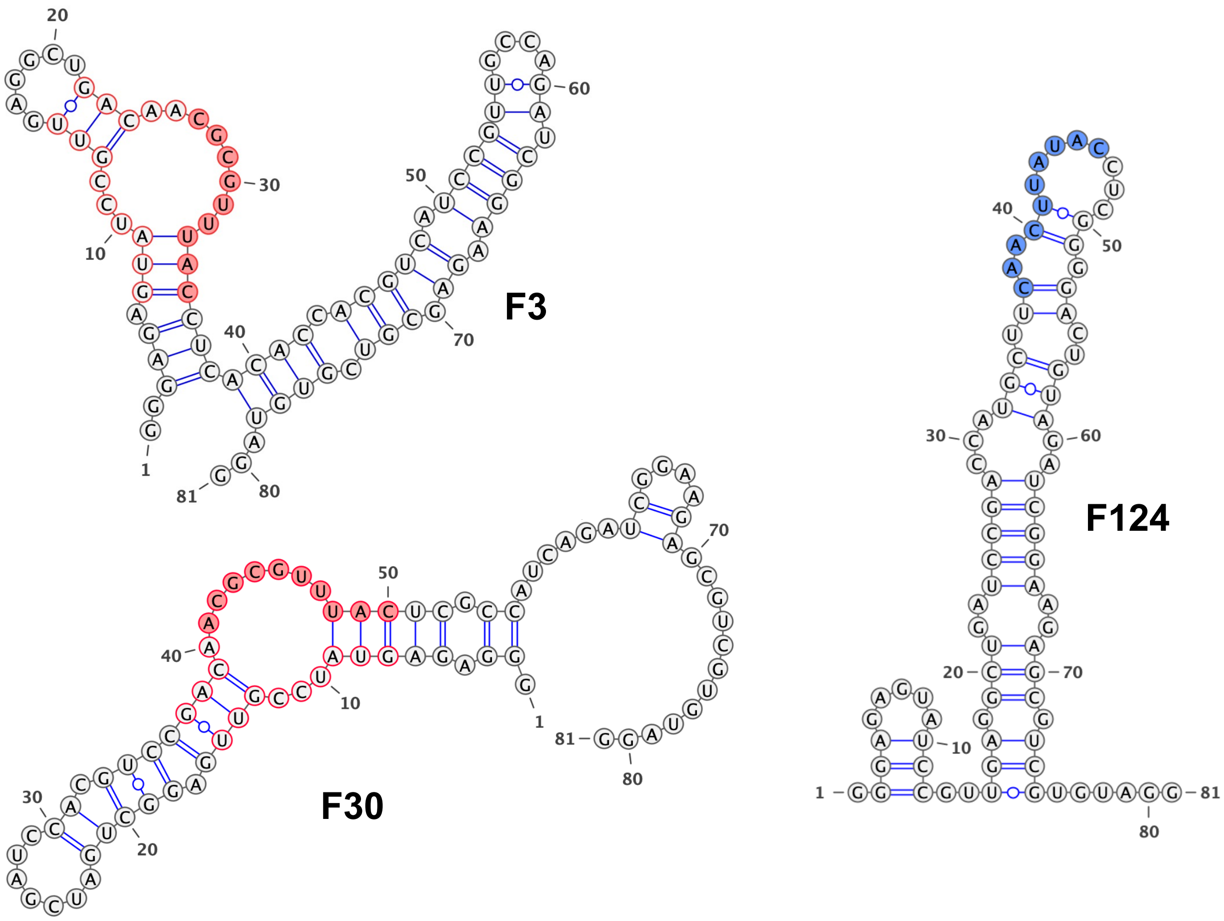

Supplementary Figure S4: Secondary structure prediction of aptamer F3, F30 and F124. Structure prediction was performed using Mfold. The bases of motif 1, present in F3 and F30 and of the motif 2 present in F124, are highlighted in red and blue, respectively.

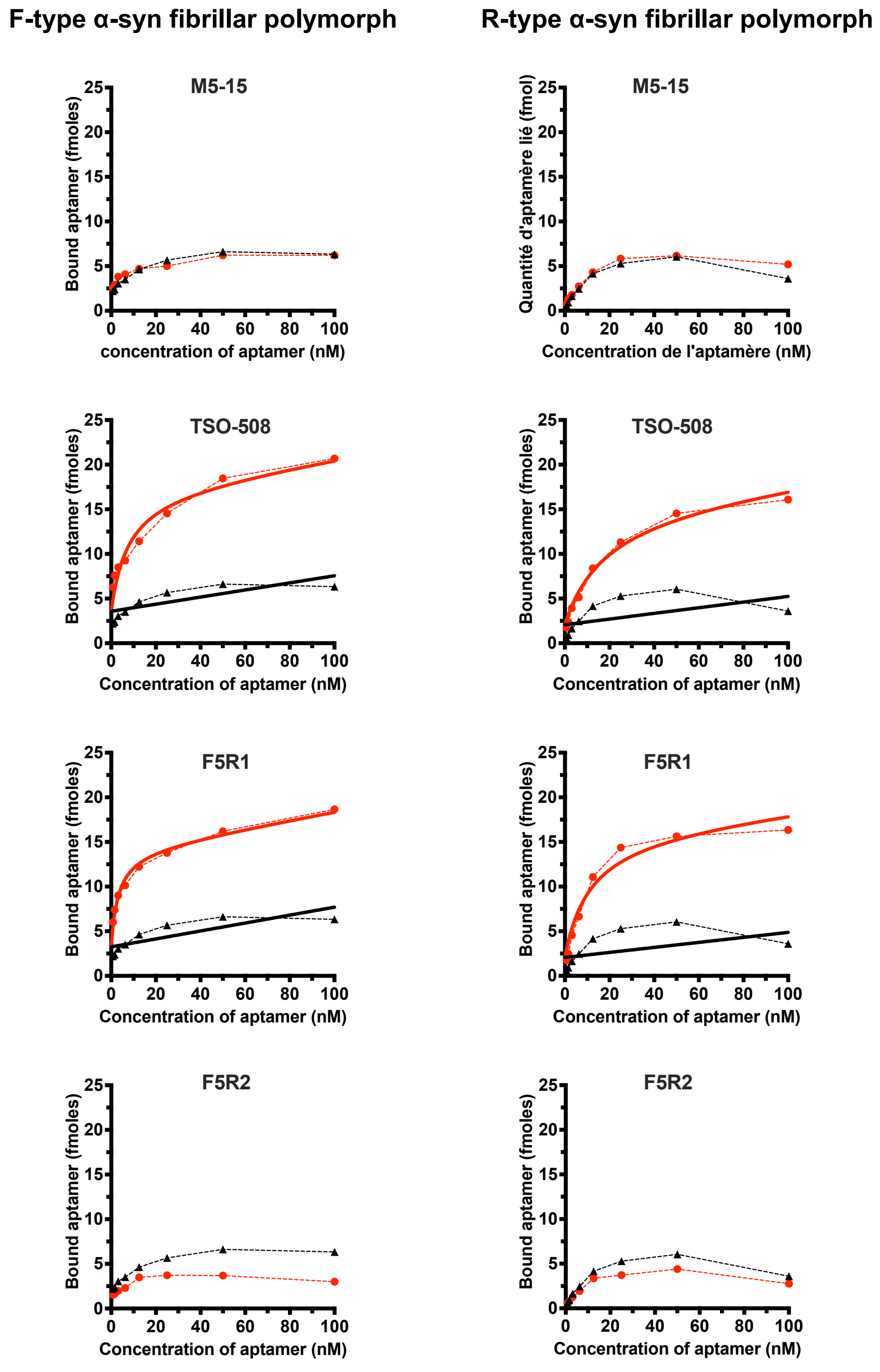

Supplementary Figure S5: Binding of DNA aptamers on F-type and R-type of α-Syn fibrillar polymorphs. Example of binding curves of different DNA aptamers against F-type (right panels) or R-type (left panels) α-Syn fibrillar polymorphs. The binding of aptamers are in red. The binding of a scramble DNA sequence was used to evaluate the nonspecific binding (black dotted curves). When the binding of aptamer was at least two times higher than that of the Scramble, the binding curves were fit using the model "One site - Fit total and nonspecific binding" from GraphPad Prism 9 to calculate the apparent Kd of the interaction.

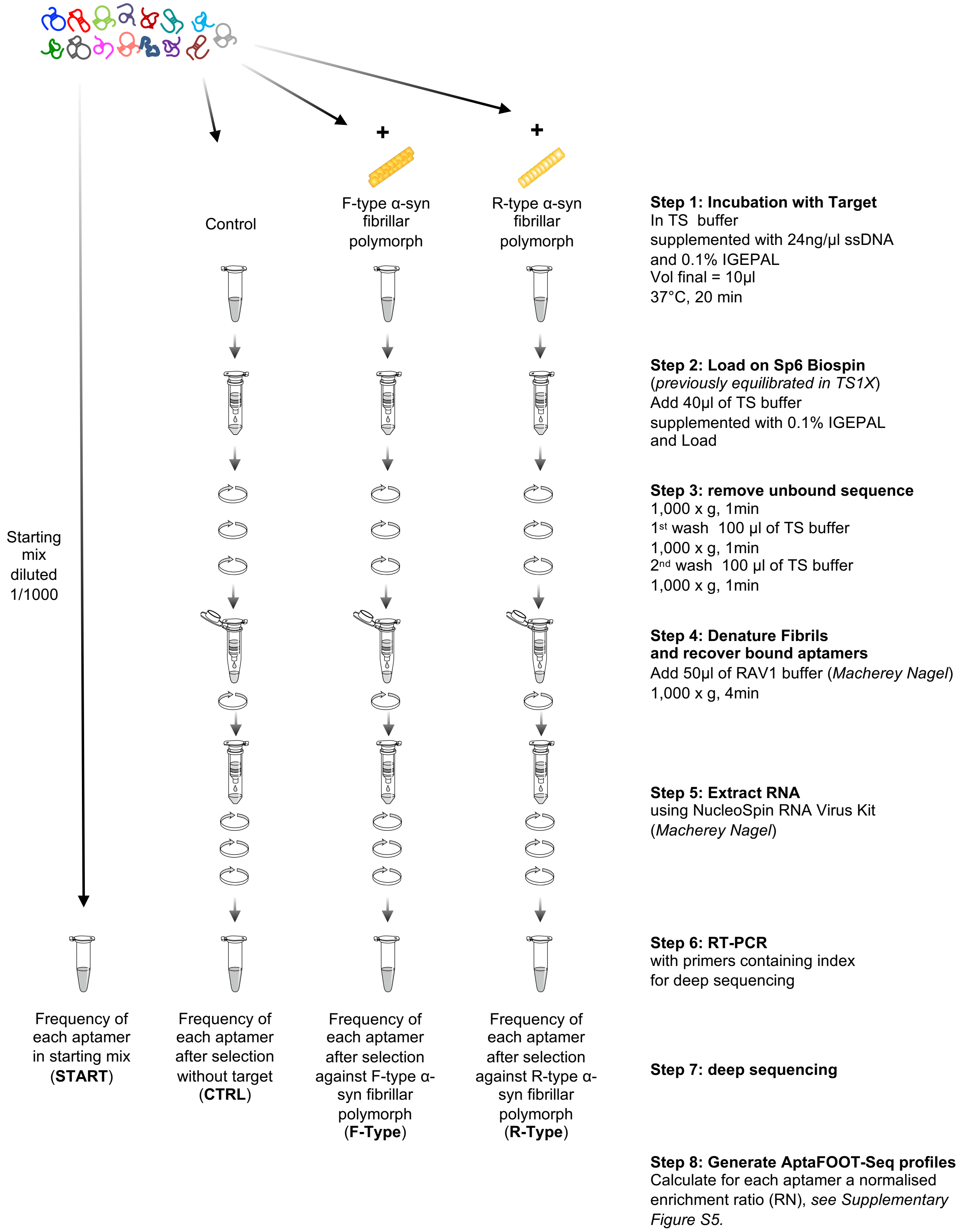

Supplementary Figure S6: Shema of the protocol of the AptaFOOT-Seq method

Frequency of each aptamer measured by NGS before selection

	START
N30	8.02%
N124	10.21%
N5	5.94%
N73	7.37%
N0	6.82%
N62	4.77%
N1	6.43%
N15	6.73%
NOAC	6.66%
N164	5.68%
N2	5.81%
N20	6.39%
N4	5.93%
N3	5.89%
Scramble	7.35%
Total	100.00%

Frequency of each aptamer measured by NGS after selection

	CTRL	F-Type	R-Type
N30	8.52%	10.57%	7.29%
N124	10.75%	14.48%	14.43%
N5	5.21%	5.35%	11.12%
N73	6.76%	14.95%	10.38%
N0	7.07%	7.47%	5.65%
N62	5.27%	3.53%	2.46%
N1	6.19%	4.20%	3.34%
N15	7.05%	14.47%	8.85%
NOAC	6.69%	5.75%	6.30%
N164	5.80%	3.57%	3.66%
N2	5.45%	2.77%	3.88%
N20	6.27%	3.38%	5.27%
N4	5.69%	3.37%	7.56%
N3	5.45%	4.04%	3.78%
Scramble	7.84%	2.11%	6.06%
Total	100.00%	100.00%	100.00%

Exemple of the calculations for N30 in CTRL:

8.52% / 8.02% = 1.06

Ratio (Frequency after selection / Frequency in starting mix)

$$r_{a,x/0} = \frac{f_{a,C=x}}{f_{a,C=0}}$$

	CTRL	F-Type	R-Type
N30	1.06	1.32	0.91
N124	1.05	1.42	1.41
N5	0.88	0.90	1.87
N73	0.92	2.03	1.41
N0	1.04	1.10	0.83
N62	1.10	0.74	0.52
N1	0.96	0.65	0.52
N15	1.05	2.15	1.31
NOAC	1.00	0.86	0.95
N164	1.02	0.63	0.64
N2	0.94	0.48	0.67
N20	0.98	0.53	0.82
N4	0.96	0.57	1.27
N3	0.93	0.69	0.64
Scramble	1.07	0.29	0.82
Total	14.96	14.34	14.60

1.06 / 14.96 = 7.1%

Frequency normalised from starting mix

$$r_{a,x} = \frac{r_{a,x/0}}{\sum_{a \epsilon A} r_{a,x/0}}$$

	CTRL	F-Type	R-Type
N30	7.1%	9.2%	6.2%
N124	7.0%	9.9%	9.7%
N5	5.9%	6.3%	12.8%
N73	6.1%	14.1%	9.6%
N0	6.9%	7.6%	5.7%
N62	7.4%	5.2%	3.5%
N1	6.4%	4.6%	3.6%
N15	7.0%	15.0%	9.0%
NOAC	6.7%	6.0%	6.5%
N164	6.8%	4.4%	4.4%
N2	6.3%	3.3%	4.6%
N20	6.6%	3.7%	5.6%
N4	6.4%	4.0%	8.7%
N3	6.2%	4.8%	4.4%
Scramble	7.1%	2.0%	5.6%
Total	100.00%	100.00%	100.00%

(7.1% - 1 / 15) / (1 / 15) = +6.45%

Normalized enrichment ratio (RN) compared to starting mix

$$RN_a = \frac{r_{a,x} - \frac{1}{n}}{\frac{1}{n}}$$

	CTRL	F-Type	R-Type
N30	6.45%	37.73%	-6.72%
N124	5.56%	48.32%	45.14%
N5	-12.05%	-5.79%	92.14%
N73	-8.03%	112.18%	44.67%
N0	3.98%	14.59%	-14.85%
N62	10.76%	-22.67%	-46.93%
N1	-3.37%	-31.63%	-46.54%
N15	5.10%	124.90%	35.01%
NOAC	0.75%	-9.64%	-2.72%
N164	2.25%	-34.34%	-33.93%
N2	-5.93%	-50.16%	-31.43%
N20	-1.61%	-44.63%	-15.37%
N4	-3.69%	-40.59%	30.96%
N3	-7.17%	-28.27%	-34.11%
Scramble	7.01%	-70.00%	-15.31%
Total	0.00%	0.00%	0.00%

Supplementary Figure S7: Example of calculation of the normalised enrichment ratio (RN). The frequency of each aptamer before and after selection is measured by NGS after RT-PCR (see Supplementary Figure S6). Although we have tried to prepare a starting mixture where each aptamer is at equimolar concentration, their frequency may be slightly different due to imperfections in pipetting or OD measurement. Therefore, we first calculate a ratio comparing the frequency of each aptamer in the test condition to its actual frequency in the starting mix. These ratios are then divided by the sum of the ratios to give the adjusted frequencies of each aptamer relative to the others normalized by its frequency in the starting mix. This normalized frequency is then compared to the expected frequency in an equimolar mixture of aptamers (in our case 1/15 because we have 15 sequences in the mix) to calculate a normalised enrichment ratio (RN) for each aptamer in the mix. The RNs are close to 0% in the CTRL condition without target, i.e. the frequency is quite the same compared to the starting mix. On the other hand, selection against F- or R-type α-syn fibrillar polymorphs induces a high increase of some aptamers in the mix and as a consequence a high decrease of others.

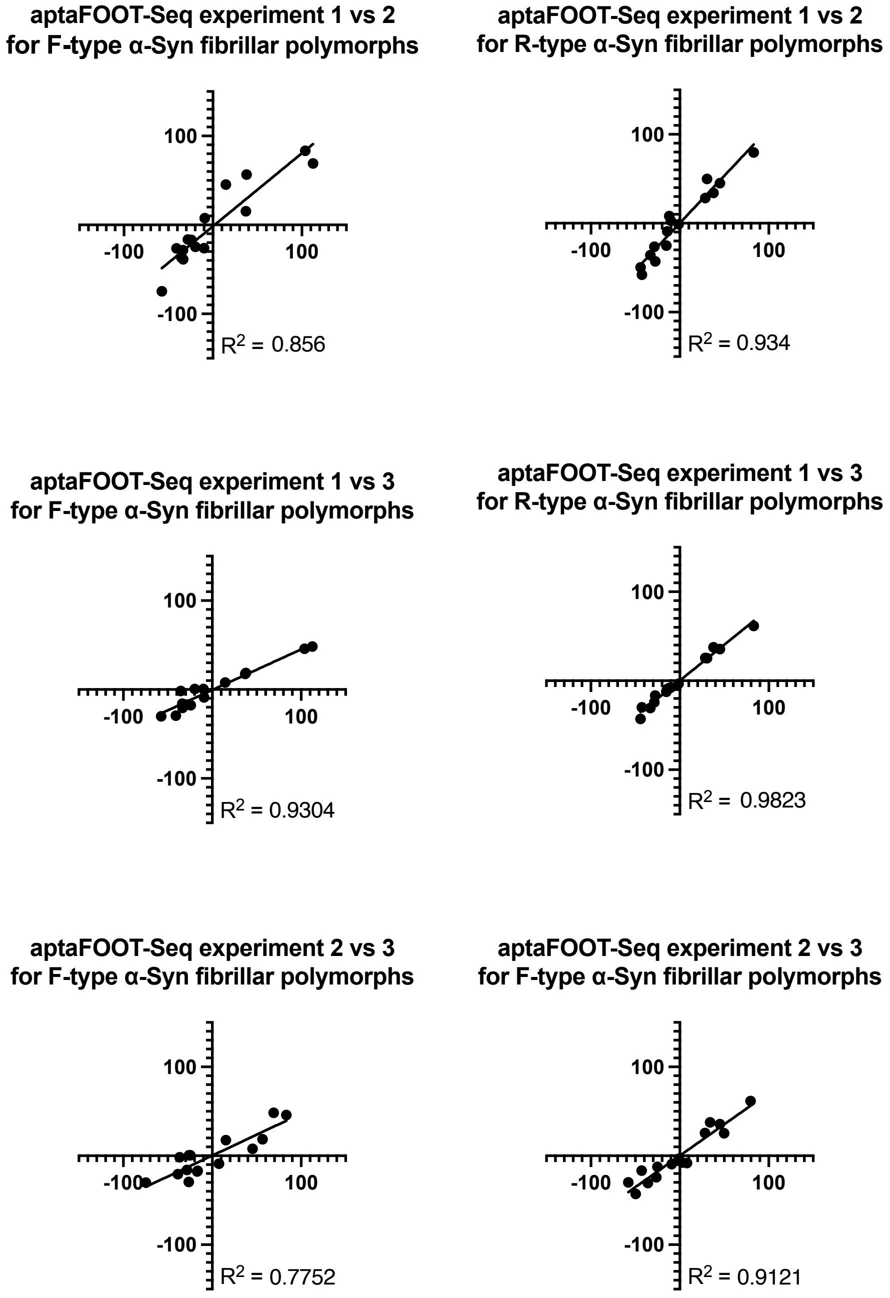

Supplementary Figure S8: Reproducibility of aptaFOOT-Seq. The reproducibility of the AptaFOOT-Seq method was evaluated by three fully independent experiments against recombinant F-type or R-type α-Syn fibrillar polymorphs (left and right, respectively). AptaFoot-Seq results show a linear correlation between experiments, demonstrating the robustness and reliability of the method with R^2 around 0.854 +/- 0.078 and 0.943 +/- 0.036 for F-type and R-type α-Syn fibrillar polymorphs, respectively.

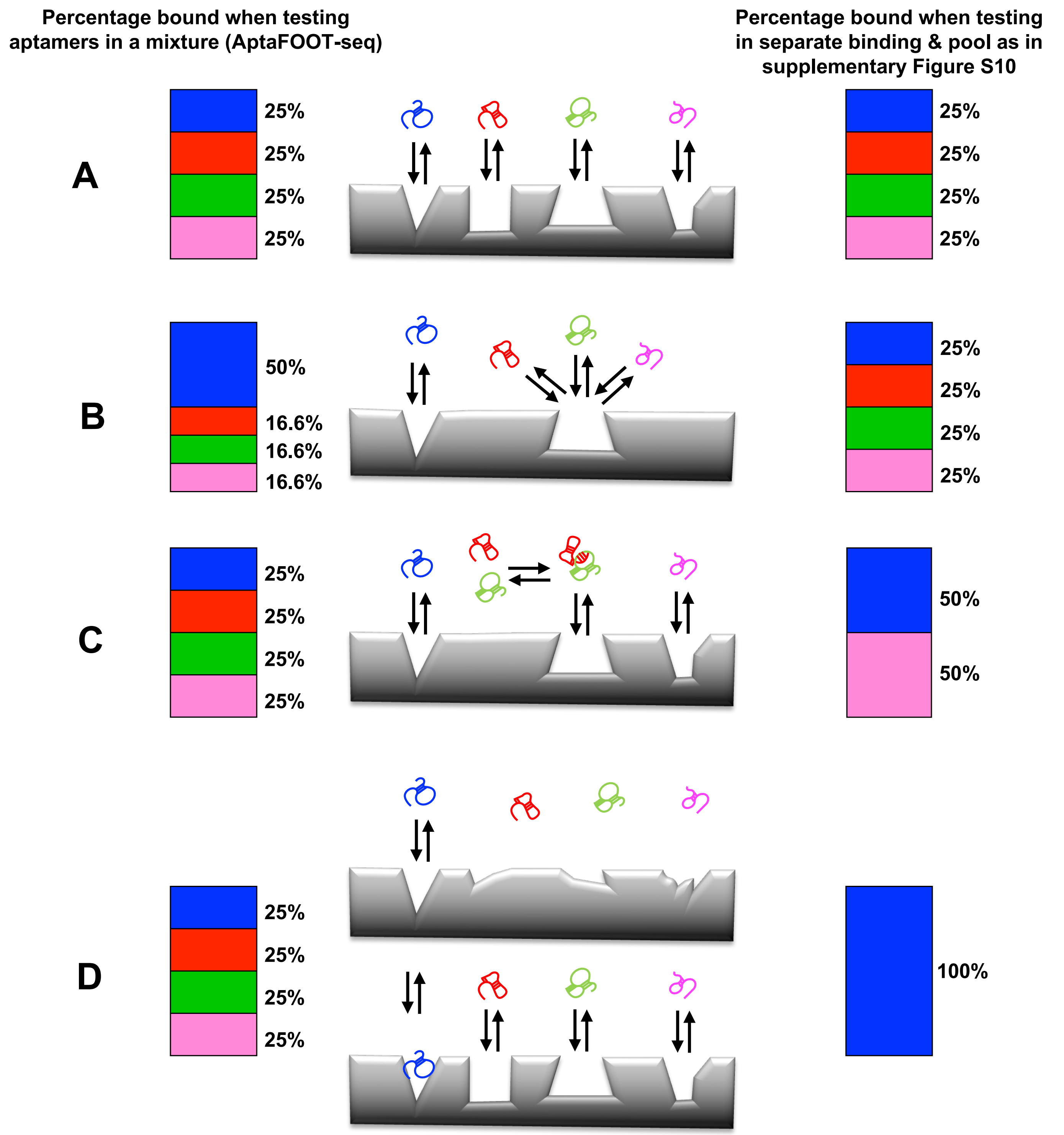

Supplementary Figure S9: Examples of different possible scenarios that may explain different binding results when aptamers are tested separately or in a mixture. For these scenarios we have assumed that all aptamers have the same affinity for their binding site. The theoretical distribution of each aptamer relative to the others when evaluated as a mixture using the AptaFOOT-Seq method or individually (as in Figure S10) is shown on the left and right, respectively. (**A**) Each aptamer have a different binding site on the target. (**B**) Three aptamers compete for the same binding site, while one aptamer (in blue) has a different binding site. (**C**) Two aptamers (in red and green) must form a complex to interact with the target, while two other aptamers (in blue and pink) have different binding sites. (**D**) One aptamer (in blue) interacts with the target, leading to a conformational change in the target that allows the binding of the three other aptamers. These models are given as examples only and do not represent an exhaustive list of all possibilities.

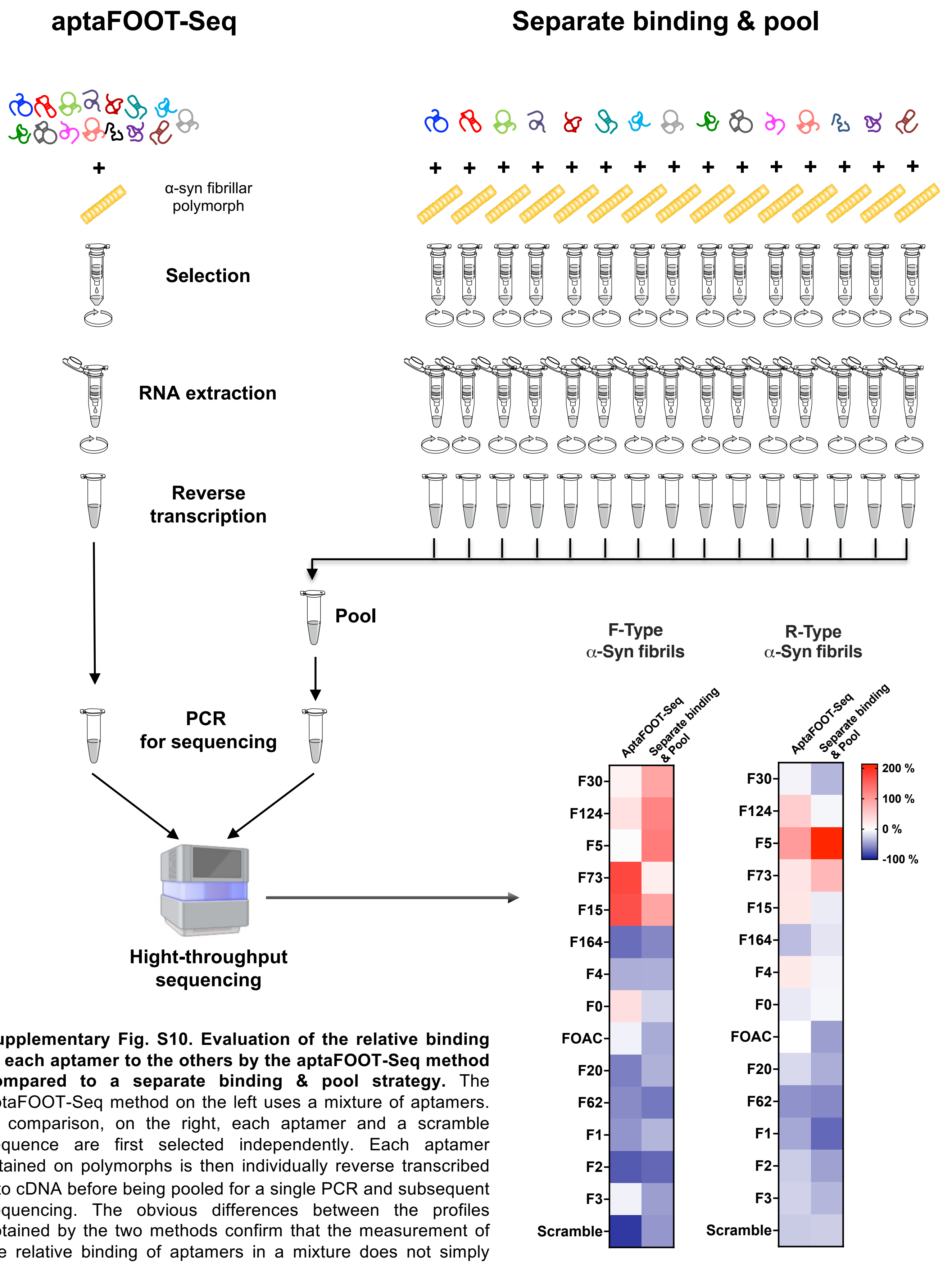

Supplementary Fig. S10. Evaluation of the relative binding of each aptamer to the others by the aptaFOOT-Seq method compared to a separate binding & pool strategy. The aptaFOOT-Seq method on the left uses a mixture of aptamers. In comparison, on the right, each aptamer and a scramble sequence are first selected independently. Each aptamer retained on polymorphs is then individually reverse transcribed into cDNA before being pooled for a single PCR and subsequent sequencing. The obvious differences between the profiles obtained by the two methods confirm that the measurement of the relative binding of aptamers in a mixture does not simply reflect the sum of their individual bindings assessed separately.
